# Data-Driven Transducer Design and Identification for Internally-Paced Motor Brain Computer Interfaces: A Review

**DOI:** 10.3389/fnins.2018.00540

**Published:** 2018-08-15

**Authors:** Marie-Caroline Schaeffer, Tetiana Aksenova

**Affiliations:** CEA, LETI, CLINATEC, MINATEC Campus, Université Grenoble Alpes, Grenoble, France

**Keywords:** brain-computer interfaces, continuous decoders, discrete decoder, dynamic/static, linear/non-linear, feature extraction

## Abstract

Brain-Computer Interfaces (BCIs) are systems that establish a direct communication pathway between the users' brain activity and external effectors. They offer the potential to improve the quality of life of motor-impaired patients. Motor BCIs aim to permit severely motor-impaired users to regain limb mobility by controlling orthoses or prostheses. In particular, motor BCI systems benefit patients if the decoded actions reflect the users' intentions with an accuracy that enables them to efficiently interact with their environment. One of the main challenges of BCI systems is to adapt the BCI's signal translation blocks to the user to reach a high decoding accuracy. This paper will review the literature of data-driven and user-specific transducer design and identification approaches and it focuses on internally-paced motor BCIs. In particular, continuous kinematic biomimetic and mental-task decoders are reviewed. Furthermore, static and dynamic decoding approaches, linear and non-linear decoding, offline and real-time identification algorithms are considered. The current progress and challenges related to the design of clinical-compatible motor BCI transducers are additionally discussed.

## 1. Introduction

Brain-Computer Interfaces (BCIs) are systems that permit their users to utilize their brain activity to control external devices without using their natural neuromuscular pathways (Leuthardt et al., 2006b; Mak and Wolpaw, 2009). BCIs are particularly being investigated for use by severely motor-impaired patients; for example, patients suffering from neuromuscular disorders such as amyotrophic lateral sclerosis (Sellers and Donchin, 2006) or patients who have sustained a spinal cord injury (Wang W. et al., 2013). BCIs aim to overcome some of the resulting motor dysfunctions by establishing a new communication pathway between the patient's brain and an effector [e.g., a robotic arm (Wodlinger et al., 2015), a speller (Yin et al., 2015), or a wheelchair (Rebsamen et al., 2006)]. The type of effector integrated into a BCI system depends on the goal of the BCI, such as: procuring patients the ability to communicate, to exert control over their environment, to displace themselves, or to recover some motor control over their limbs (Mak and Wolpaw, 2009).

**Figure 1:**
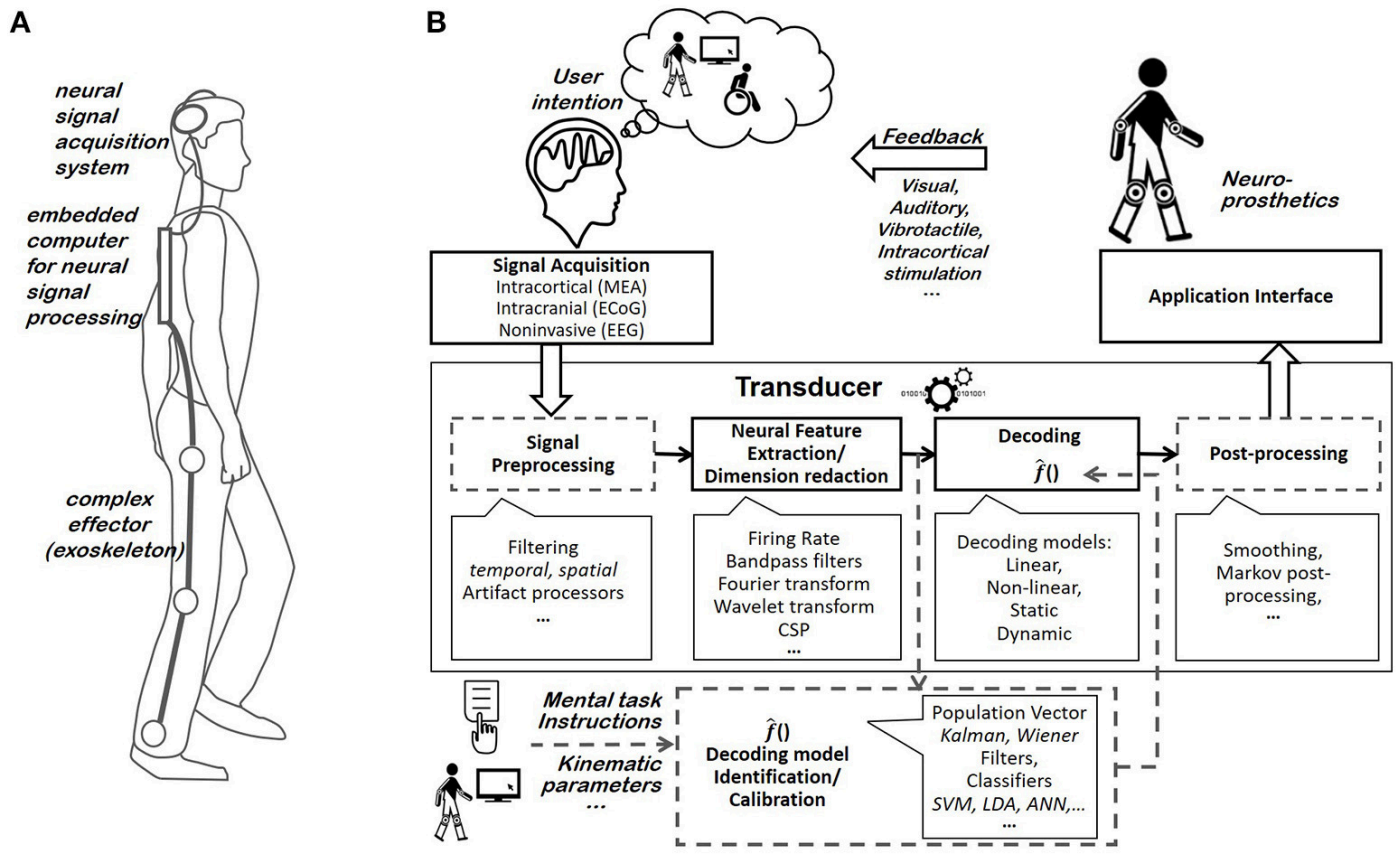
**(A)** Example of an internally-paced motor BCI system. The user generates brain patterns specific to the movement that he or she intends to execute. After having been acquired by the acquisition system, his or her neural signals are processed and converted into commands used to control the effector's state (e.g., its position, velocity, aperture, etc.). **(B)** BCI components. Most BCI systems include a neural signal acquisition system, a transducer, an effector, and a feedback system. The transducer is typically composed of a neural feature extraction block and a decoder, and it optionally includes a pre-processing and a post-processing block. It is generally adapted to the user. The decoder's user-specific identification/calibration block is fed with both neural features and the corresponding mental task or kinematic parameters (e.g., instructions provided to the patient).

The present review focuses on motor BCIs, which endeavor to restore limb mobility in severely motor-impaired patients by providing them with control over orthoses or prostheses (Figure 1A). While motor BCIs rely on the same components as other BCIs (e.g., BCIs which offer patients cursor control for communication or environmental purposes), they present particular challenges due to specific constraints associated with the control of physical effectors. A high accuracy is particularly required to control prostheses or orthoses. Consequently, research on motor BCIs has only recently taken off, while communication BCIs have already been commercialized. The first demonstrations of the feasibility of neurally driven cursor control (Vidal, 1977) were quickly followed by studies that were completed with invasive neural signals and suggested that complex neural control over protheses or orthoses could be achieved by exploiting both the natural encoding of trajectory kinematics in neuronal activity (Georgopoulos et al., 1982) and brain plasticity (Schmidt et al., 1978). By the early-2000s, trajectory-tuned features had not been profitably exploited in motor BCI systems and complex effector control had not yet been achieved. Furthermore, only simplistic prosthetic control based on motor imageries detected through non-invasive neural signals had been reported (Guger et al., 1999; Pfurtscheller et al., 2000). These motor BCIs implemented decoding strategies similar to the strategies that are frequently utilized for neural control for communication or environmental control (e.g., exploitation of differences between the neural patterns generated by a set of discrete cognitive tasks). Complex motor effector control began to emerge at the same time (Wessberg et al., 2000; Taylor et al., 2002; Carmena et al., 2003). Finally, the first demonstrations of complex prosthesis control were achieved in 2006 (Hochberg et al., 2006). Control complexity has since steadily improved and it now relies on much more complex decoding strategies (Hochberg et al., 2012; Wodlinger et al., 2015).

BCI systems are based on the interpretation of brain activity patterns. Specific and measurable patterns must be generated by the user's brain to trigger the execution of a particular movement by the prosthesis or orthosis integrated into the motor BCI system. Motor BCIs exploit either externally- or internally-paced neural activities. Externally-paced brain patterns are responses that are evoked by a visual, auditory or somatosensory stimulus (Evoked Potential). By contrast, internally-paced BCIs rely on the brain patterns that are voluntarily elicited by users, such as Slow-Cortical Potentials (SCP) and Sensorimotor Rhythms (SMR) (Waldert et al., 2009). BCI systems rely on several components to translate internally- or externally-paced brain patterns into prosthesis or orthosis movements, including the cerebral signal acquisition system, the transducer permitting to translate brain activity measurements into estimates of the user's intention, the controlled effector and the feedback provided to the user (Schwartz et al., 2006) (Figure 1B).

**Figure 2:**
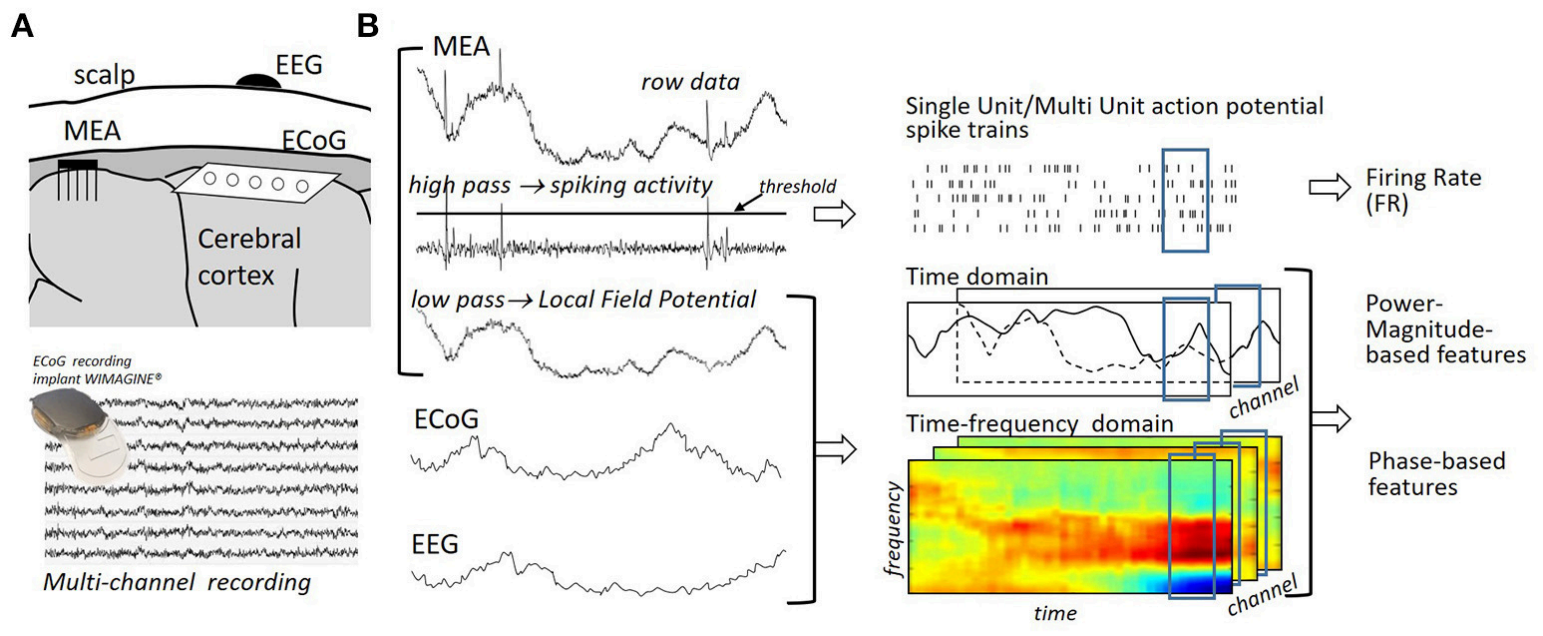
**(A)** Multichannel acquisition systems mostly used in motor BCIs. Invasive intracortical Microelectrodes Arrays (MEA), intracranial and noninvasive EEG arrays, which measure the electrical activity generated by neurons, correspond to different trade-offs between invasiveness and spatial resolution. Sensors are located at a distance which ranges from a few μm (MEA) to several cm (EEG) from the cortical neurons generating the currents of interest (Waldert et al., 2009). This distance impacts the size of the neuronal population observed by sensors and, therefore, the spatial resolution of the acquired signals. **(B)** Local Field Potential (LFP) and Single Unit/Multi Unit Action Potential (SUA/MUA) spike trains are extracted from MEA recordings by means of low/high pass filtering. Spike counts in spike trains result in Firing Rate (FR) neural features. LFP, ECoG, EEG multi-channel recordings are considered in time/time-frequency domains to extract power-, magnitude,- or phase-based features.

**Acquisition system**: The acquisition system is used to sample, amplify and digitize a measure of the user's cerebral activity (Homer et al., 2013). While the exploitation of magnetic or metabolic neural signals is being investigated by several teams (e.g., Naseer et al., 2014; Hong et al., 2015), most motor BCIs currently rely on the measure of electrophysiological signals; that is, on signals originating from the electrical currents generated by neurons (Mak and Wolpaw, 2009). The use of Microelectrodes Arrays (MEA) (Hochberg et al., 2012; Collinger et al., 2013; Wodlinger et al., 2015), Electrocorticographic (ECoG) (Schalk et al., 2008; Vansteensel et al., 2010; Kellis et al., 2012; Wang W. et al., 2013; Fifer et al., 2014; Kapeller et al., 2015) or Electroencephalographic (EEG) arrays (Pfurtscheller et al., 2000; Onose et al., 2012; Baxter et al., 2013) have been reported for electrophysiological signal acquisition in motor BCI systems (see Figure 2A). These devices measure electrical fields at different distances from the cortex and, therefore, exhibit different degrees of invasiveness and spatial resolutions (Schwartz et al., 2006). MEAs are invasive arrays that directly sample neurons' electrical activity from within the brain (intracortical recordings) (Homer et al., 2013). Preprocessing permits us to extract three signals from this electrical activity, namely: Single-Unit Activity (SUA), Multi-Unit Activity (MUA) and Local Field Potentials (LFP) (Waldert et al., 2009). While MUA and SUA signals reflect the spiking activity of the few neurons located in the immediate vicinity of the electrode's tip (Leuthardt et al., 2006a; Homer et al., 2013), LFPs measure the superposed activity of a small population of neurons located in the neighborhood of the electrode's tip (Leuthardt et al., 2006a; Homer et al., 2013). ECoG arrays acquire the cerebral activity at the surface of the brain (Mak and Wolpaw, 2009). In contrast to MEAs, ECoG arrays are said to be semi-invasive (Rak et al., 2012). The EEG is a non-invasive record of the brain's electrical fields (Berger, 1929). In general, it mostly reflects extracellular currents that are generated by synchronously activated group of neurons (mainly from pyramidal neurons), which are recorded by channels placed on the scalp (Lopes da Silva, 2013).

**Transducers**: The BCI transducer translates brain activity measurements into estimates of the user's intention. Transducers are generally composed of several signal processing blocks (Bashashati et al., 2007a). A first, optional step consists of enhancing the raw cerebral signals. Features specifically related to the user's intentions are then extracted from the cerebral signals (Mak and Wolpaw, 2009). A decoder, also referred to as “translation algorithm” (Yuan and He, 2014) or “feature translator” (Bashashati et al., 2007a), interprets the brain features and issues an estimate of the user's intention. Discrete decoders (i.e., classifiers) are used to estimate discrete user intentions (e.g., movement toward the right vs. movement toward the left). Meanwhile, continuous decoders permit to decode continuous user intentions (e.g., 3D position or velocity). After being optionally enhanced by post-processing methods (Bashashati et al., 2007a), intention estimates are conveyed to the effector's controller.

**Effectors:** Custom and commercialized hand (Pfurtscheller et al., 2000; Murguialday et al., 2007; Chen et al., 2009; Ortner et al., 2011; Bundy et al., 2017), upper- (Bougrain et al., 2012; Webb et al., 2012; Baxter et al., 2013; Collinger et al., 2013; Wang W. et al., 2013; Morinière et al., 2015; Wodlinger et al., 2015), and/or lower limbs (Gancet et al., 2012; Do et al., 2013a; Eliseyev et al., 2014; Nicolelis, 2014; Kwak et al., 2015; López-Larraz et al., 2016) orthoses and prostheses have been neurally manipulated by motor BCI users. Early results on the utilization of Functional Electrical Stimulation (FES), which consists of stimulating the user's muscles, have been presented in a few studies (King et al., 2015; Bouton et al., 2016; Vidaurre et al., 2016) Finally, virtual effectors—such as cursors (Taylor et al., 2002; Leuthardt et al., 2006a; Kim et al., 2008; Simeral et al., 2011) or simulated robotic arms in virtual reality environments (Ifft et al., 2013; Wang W. et al., 2013; Wodlinger et al., 2015)—have regularly been exploited to facilitate early training phases. Cursor-control BCI studies have, therefore, been included in the present review.

**Feedback:** Volitional motor control is permitted by the perception and exploitation of feedback regularly delivered to users through different afferent pathways (Suminski et al., 2010), such as proprioceptive, visual, auditory or tactile feedback. In the vast majority of motor BCIs, the users are exclusively provided with visual feedback about the transducer output [e.g., MEA- (Kim et al., 2011; Hochberg et al., 2012; Collinger et al., 2013; Wodlinger et al., 2015), ECoG- (Vansteensel et al., 2010; Kellis et al., 2012; Milekovic et al., 2012; Yanagisawa et al., 2012; Wang W. et al., 2013), and EEG-based clinical trials (Wolpaw and McFarland, 2004; Yuan et al., 2007; McFarland et al., 2010; Doud et al., 2011; LaFleur et al., 2013)]. The combination of several types of feedback (e.g., visual and kinesthetic feedback in Suminski et al., 2010; Bundy et al., 2017) has been shown to facilitate upper-limb prosthesis control (Suminski et al., 2010). Haptic feedback has additionally been used to improve neural control over a hand prosthesis in (Murguialday et al., 2007) and a few teams have completed preliminary studies (Cincotti et al., 2007) or cursor control experiments (Chatterjee et al., 2007) with vibrotactile feedback. Finally, the feasibility of intracortical stimulation-based feedback has been demonstrated (O'Doherty et al., 2011). Because feedback is regularly delivered to users when BCI systems are deployed, BCI users are said to be provided with so-called closed-loop control over the effector.

The present article reviews the transducers that have been integrated into MEA-, ECoG-, and EEG-based motor BCI systems. In particular, user-specific data-driven transducers are surveyed. However, recent efforts to develop user-independent transducers (Fazli et al., 2009; Gaur et al., 2016) are not reviewed, and attempts at discarding transducer training and exclusively relying on user training (Ganguly and Carmena, 2009) are not exhaustively debated. Finally, transducers designed for externally-paced motor BCIs are not included.

The rest of this review is organized as follows. The remainder of the introduction is devoted to a presentation of the user- and decoder-adaptation strategies utilized to reach a high consistency between user intentions and transducer's estimates. It then includes insights on the two decoding approaches that are most frequently exploited by motor BCI transducers, namely: the biomimetic and mental-task approaches. While the biomimetic approach relies on the natural mapping between neural patterns and limb movements, a new mapping is learned by users of mental-task motor BCIs. The two main components of the transducer, namely the feature extraction block and the decoder, are then thoroughly reviewed in the second and third section. Finally, the transducer-specific challenges that remain to be addressed for motor BCIs to fully benefit motor-impaired patients are discussed.

### 1.1. Designing user-specific, data-driven decoders for motor BCIs

Motor BCI systems benefit patients if the decoded actions reflect the user's intentions with a fidelity enabling them to efficiently interact with their environment with the controlled effector. Transducer design consists of constructing and adapting the signal translation blocks to reach a high decoding accuracy. Data-driven user-specific decoder design, which is more specifically considered in the present review, generally relies on two processes to reach a high consistency (accuracy) between user intention and transducer's output: decoder adaptation (i.e., identification) and/or user adaptation (i.e., training) (McFarland and Wolpaw, 2011).

#### 1.1.1. Decoder adaptation

Decoder identification is performed by analyzing a dataset of simultaneously acquired neuronal signals and intended effector movements. It is carried out after a decoder structure has been selected on the basis of preliminary studies and it consists of tuning this decoder over this training dataset. This tuning phase is also referred to as decoder “adaptation” (McFarland and Wolpaw, 2011), “learning” (e.g., Hudson and Burdick, 2007), “training” (e.g., Ifft et al., 2013) or “calibration” (e.g., Jarosiewicz et al., 2013). The training dataset is often collected during open-loop acquisition sessions; that is, sessions during which the future BCI user is not given feedback on the output of the BCI transducer but is generally cued to repeatedly generate action-specific patterns.

Because of a context difference, open-loop neural patterns differ from closed-loop patterns (Leuthardt et al., 2006a; Jackson and Fetz, 2011; Jarosiewicz et al., 2013). Performance drops are regularly observed when an open-loop decoder is applied during closed-loop experiments (Tillery et al., 2003). Decoders trained on data acquired during closed-loop control sessions have more specifically been shown to outperform decoders calibrated with open-loop data (Jarosiewicz et al., 2013).

However, the neural signals processed by the transducer only reflect the possibly superposed activity of neurons localized in restricted parts of the users' brain areas involved in motor control and they are liable to be substantially corrupted by noise. User intentions estimated from neural signals thus exhibit limited accuracy. This limitation makes user training indispensable.

#### 1.1.2. User adaptation

**Figure 3:**
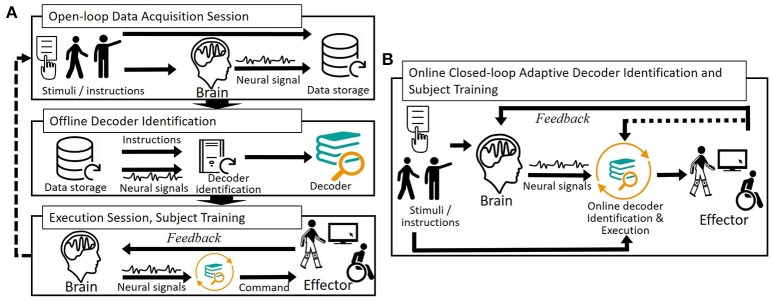
Open- and closed-loop user-specific decoders identification and subject training. **(A)** Decoder identification is performed offline by analyzing a dataset of simultaneously acquired neuronal signals and intended movements (open-loop data acquisition session). The resulting decoder is applied online on the user's neural signals so that he or she can train; that is, adapt his or her neural patterns to the imperfect decoder. Because the user progressively modifies his or her brain patterns, one or several blocks of decoder re-identification can be completed. **(B)** Simultaneous decoder and user adaptation using adaptive/incremental learning algorithms permits to directly identify a decoder associated with closed-loop neural patterns.

Training permits BCI users to adapt to imperfect decoders. Thanks to the feedback that is provided to them, the users are able to assess the difference between their intention and the transducer's output, and progressively learn to reduce it by modifying their brain patterns (Figure 3A). User adaptation exploits brain plasticity (McFarland and Wolpaw, 2011); that is, the brain ability to reorganize to learn new tasks.

BCIs based on user adaptation have only been explored in MEA-based preclinical studies (Ganguly and Carmena, 2009). In particular, the limits of brain plasticity have been investigated in a few studies (Ganguly and Carmena, 2009; Sadtler et al., 2014). These findings suggest the limitations of users' adaptation ability and they support the relevance of the combination of decoder and user adaptation.

#### 1.1.3. Combining decoder and user adaptation

Several strategies have been reported to combine decoder and user adaptation, the simplest being to let the user train after an initial open-loop decoder initialization. More complex strategies consist of re-identifying the decoder during closed-loop BCI sessions (Gilja et al., 2012; Hochberg et al., 2012) (Figure 3B). One or several (Hochberg et al., 2012; Wang W. et al., 2013) blocks of successive decoder and user adaptation have particularly been reported in preclinical and clinical motor BCIs (Shenoy and Carmena, 2014) (e.g., Wang W. et al., 2013; Wodlinger et al., 2015).

### 1.2. Decoding strategies

Different decoding (mapping) strategies are used to provide users with control over orthoses or prostheses. They are thought to impact the user's ability to control multi-limb effectors along multiple Degrees of Freedom (DoF) and also the mental load associated with neural control.

#### 1.2.1. Direct decoding: biomimetic kinematic decoding

Biomimetic decoding exploits the mapping which relates neuronal activity to limb movement before the patient began to suffer from motor disabilities; that is, it uses the activity of neurons naturally devoted to the control of a specific limb to compute the commands sent to the corresponding prothesis or orthosis. These decoders are often referred to as “direct” decoders [“direct motor Brain Machine Interfaces” (Waldert et al., 2009), “direct mapping” (Degenhart et al., 2018)]. Most biomimetic decoders are kinematic. They directly extract the effector's continuously-valued kinematic parameters from the corresponding neural signals, such as the position or velocity of an orthosis endpoint.

Neural features correlated with the kinematic parameters of the intended effector movement were first discovered in the spiking activity of monkeys performing reaching movements (Georgopoulos et al., 1982). Firing rate tuning has since been extended to other trajectory characteristics (Scott, 2008). Correlations have particularly been observed between motor neurons' spiking activity and hand speed (Churchland et al., 2006), position (Paninski et al., 2004; Wang et al., 2007), velocity (Paninski et al., 2004; Wang et al., 2007) and acceleration (Ashe and Georgopoulos, 1994), joint motion (Vargas-Irwin et al., 2010), or muscle activation (Koike et al., 2006). Neuron tuning has additionally been found to persist when humans with tetraplegia attempt to execute arm movements (Hochberg et al., 2006), suggesting that the utilization of kinematic decoders is achievable by motor-impaired patients.

While tuned features were discovered in neurons' spiking activity, an increasing number of studies have disclosed the existence of features correlated with trajectory kinematics in the activity of neuronal populations (Waldert et al., 2009), such as in LFP (Mehring et al., 2003, 2004), EEG (Waldert et al., 2008; Bradberry et al., 2010), ECoG (Gunduz, 2008; Ball et al., 2009; Anderson et al., 2012; Nurse et al., 2015a), or MEG (Waldert et al., 2008; Bradberry et al., 2009) signals.

To date, biomimetic kinematic transducers have mainly been embedded in MEA-based BCI systems (e.g., Hochberg et al., 2012; Collinger et al., 2013; Ifft et al., 2013; Wodlinger et al., 2015), which have permitted users to achieve accurate MEA-driven neural control over multiple degrees of freedom in clinical studies (Collinger et al., 2013; Wodlinger et al., 2015). Neuronal population features tuned to kinematic parameters have principally been exploited in offline analyses, and have led to fine movement reconstruction from LFP (Mehring et al., 2003), ECoG (Chao et al., 2010; Shimoda et al., 2012; Bundy et al., 2016), and EEG signals (Bradberry et al., 2010; Ofner and Müller-Putz, 2012). Online 2D control based on kinematic decoding has additionally been reported in primates implanted with ECoG arrays in Marathe and Taylor (2013). Finally, while the feasibility or use of biomimetic kinematic transducers has mostly been investigated for upper-limb effectors, the results suggest that they may also be considered for MEA-driven lower-limb effector control (Fitzsimmons, 2009; Ma et al., 2017).

Although most kinematic decoders are biomimetic, another type of MEA-based kinematic decoders has been explored by a few teams, namely: biofeedback decoders (Ganguly and Carmena, 2009; Sadtler et al., 2014). Biofeedback decoders also focus on the activity of motor neurons but they rely on pure user training rather than on the exploitation of the user's natural map between neuronal activity and limb kinematic parameters. While the respective relevance of biomimetic and biofeeback decoders is still unclear, particularly in terms of training duration (Jackson and Fetz, 2011; Carmena, 2013), most MEA-based motor BCIs rely on biomimetic kinematic decoders that are optimized through decoder and user adaptation (Hochberg et al., 2012; Wodlinger et al., 2015).

#### 1.2.2. Indirect decoding: mental-task decoding

A second approach consists in using the activity elicited in brain areas that were not exclusively devoted to the control of the limb of interest. For example, the brain patterns that are used to control the prosthesis or orthosis movements are elicited by mental tasks, such as motor imageries (somatotopic remapping), and cognitive tasks. “Mental-task” decoding (Waldert et al., 2009) or “abstract” mapping (Degenhart et al., 2018) are some of the terms that have been used in the literature to refer to BCIs based on unnatural motor imageries and cognitive tasks or strategies. Various mental tasks have been used to elicit intention-specific and distinguishable brain patterns for neural control in motor BCI systems (Waldert et al., 2009). Motor imageries associated with different limbs (e.g., tongue, foot, right arm, left arm etc.) are routinely exploited in motor BCIs based on analog neural population signals (Waldert et al., 2009) because they generate patterns which are spatially distinguishable at a macroscopic scale (Waldert et al., 2009), such as in EEG (McFarland et al., 2010) and ECoG (Wang W. et al., 2013) signals. Several studies have additionally focused on the discrimination between cognitive tasks (Penny and Roberts, 1999; Curran et al., 2004); that is, tasks not associated with patterns generated in the motor cortex (Jackson and Fetz, 2011). Given that both motor imageries and cognitive tasks can be utilized in motor BCI systems, studies relying on either type of mental tasks have been included in the present review.

Mental-task decoders are either continuous (Wang W. et al., 2013) or discrete (Bhattacharyya et al., 2015; Hortal et al., 2015). In the latter case, discrete- or continuously-valued effector kinematic commands are subsequently inferred from the discrete or probabilistic output (Milekovic et al., 2012) of the mental-task decoder. A mental task is, for example, associated with a movement toward a specific direction at a fixed speed, or movement velocity is proportional to specific features detected during a screening procedure as being easily modulated by the user.

#### 1.2.3. Applicability and relevance of biomimetic kinematic and mental-task decoders

Although further studies are required to assess the limits of biomimetic kinematic control, it is often presented as a profitable feature for motor BCI systems (Chin et al., 2007; Yuan and He, 2014). The associated neural control is expected to be more intuitive (Schalk et al., 2007; Pistohl et al., 2008; Ashmore et al., 2012; Nurse et al., 2015a) and more precise (Chin et al., 2007; Nurse et al., 2015a) than mental-task-based neural control, thus reducing the user's mental load (Yuan and He, 2014) and the user's necessary training duration (Waldert et al., 2009).

However, these expected advantages of biomimetic kinematic decoding are likely to be conditioned on the feasibility of estimating highly accurate kinematic parameters from neural features. It has, for example, been shown that the ability of users to execute reaching movements is degraded when estimated positions or velocities are not sufficiently correlated with the user's intentions; for example, when correlation is equal or inferior to 0.75 or 0.5 in the case of position and velocity decoding, respectively (Marathe and Taylor, 2011). This finding suggests that biomimetic kinematic neural control of the effector is profitable only when the estimated kinematic parameters are highly accurate; that is, when highly tuned features can be extracted from neural signals. While high correlations between real and estimated kinematic parameters are regularly reported in MEA-based trajectory reconstructions [e.g., coefficient of determination of 0.76 and 0.83 when regressing 3D position and velocity on neural signals (Wang et al., 2007)], the feasibility of kinematic ECoG- and EEG-driven control remains to be clearly established. Offline reconstruction of upper-limb trajectories has been reported in EEG- (Waldert et al., 2008; Bradberry et al., 2010; Jerbi et al., 2011; Úbeda et al., 2017) and ECoG-based (Gunduz, 2008; Ball et al., 2009; Anderson et al., 2012; Bundy et al., 2016) kinematic decoder feasibility studies. The reported Pearson' Correlation Coefficients (PCC) between true and estimated trajectories are, however, lower than those achieved with MUA/SUA or LFP signals. Average PCCs inferior to 0.6 and 0.3 were reported for ECoG- and EEG-based estimation of 3D positions or velocities, respectively (Bundy et al., 2016; Úbeda et al., 2017).

Mental-task decoders have the advantage of remaining efficient when the acquired signals exhibit a limited spatial resolution; that is, when they reflect the activity of neurons located in a relatively large cortex area around the sensor. Non-invasive and semi-invasive neuronal population recording technologies are not capable of recording cortical activity at the same spatial resolution as intracortical MEAs (millimeter and centimeter scale for ECoG and EEG recordings, respectively, Schalk and Leuthardt, 2011; Buzsáki et al., 2012). Most non-invasive acquisition systems are thus associated with mental-task transducers (Waldert et al., 2009; Milan and Carmena, 2010). For example, 3D EEG neural control over a quadcopter has been achieved in LaFleur et al. (2013) by using volitional modulation of patterns elicited via motor imagery. Similarly, EEG-based neural control permitted users to perform 3D reaching movements in a virtual space in McFarland et al. (2010). Mental-task decoders have also been used for cursor or prosthesis control from ECoG signals (Wang W. et al., 2013) and, occasionally, from SUA/MUA signals (Hochberg et al., 2006). More specifically, most online ECoG-driven motor BCI studies have been completed with mental-task decoders (Leuthardt et al., 2004, 2006a, 2011; Schalk et al., 2008; Vansteensel et al., 2010; Degenhart et al., 2018).

However, the complexity of the control tasks achieved with mental-task decoders (e.g., 3D control; LaFleur et al., 2013) remains lower than the one reported with biomimetic kinematic decoders (e.g., 10D continuous control; Wodlinger et al., 2015).

## 2. Feature extraction

Once acquired, neural signal are processed within the transducer. An optional first step consists in preprocessing these signals (Schalk et al., 2007; Galán et al., 2008; Lew, 2012; Onose et al., 2012; Shin et al., 2012; Hammer et al., 2013); for example, to reduce or discard ocular or cardiac artifacts and/or to increase the Signal-to-Noise-Ratio. Manual (Kubánek et al., 2009; Flint et al., 2012) or automatic (López-Larraz et al., 2016) artifact rejection is mainly performed for offline cleaning of training data before model identification. Meanwhile, artifact removal approaches aim to correct rather than rejecting neural signals corrupted by artifacts and are suited for online application. Temporal filtering (Sadeghian and Moradi, 2007; Herman et al., 2008), linear regression (Ferreira et al., 2008), spatial filtering (Brunner et al., 2007) or alternative strategies (Eliseyev and Aksenova, 2014; Daly et al., 2015; Foodeh et al., 2016) are some of the preprocessing methods which have been applied in motor BCI systems. During a second step, which is carried out by the majority of the transducers designed for motor BCIs, neural features are extracted from the raw or pre-processed signals.

### 2.1. Neural feature extraction

Feature extraction permits us to build a new representation of neural signals, bringing out the signals' informative attributes and discarding redundant or irrelevant characteristics. The utilization of several methods has been reported for the extraction of neural features in motor BCIs. Their applicability depends on the considered neural signals; for example, spike counts are exclusively extracted from MEA signals while time-frequency features are generally used to characterize analog neural population signals (Figure 2B).

#### 2.1.1. Spike count

MEAs permit the extracellular recording of action potentials (spikes) that are mainly generated by neurons located within the cerebral cortex. Most MEA-based motor BCIs relied on the analysis of the activity of individual neurons rather than of populations of neurons; that is, they exploited characteristics of MUA or SUA signals (e.g., Collinger et al., 2013; Wodlinger et al., 2015). Spike detection, which permits us to access MUA signals, is first performed by thresholding high-pass filtered neural signals (>300 Hz, Waldert et al., 2009). Manual or automatic spike sorting is often carried out to obtain SUA signals; that is, to decouple the activity of each observed unit or of groups of units (Kemere et al., 2004; Hochberg et al., 2006; Ganguly et al., 2009; Li et al., 2011; Wodlinger et al., 2015). The detected spikes are generally characterized by their number of occurrence in short time bins (firing rate, see Figure 2). Fine control over prostheses has been achieved by users in several spike count-based motor BCIs; see, for example, Hochberg et al. (2012), Collinger et al. (2013), and Wodlinger et al. (2015). Alternative spike-base d features have additionally been exploited in motor BCIs. Control based on the point process filtering of instantaneous spiking events has, for example, been reported in Shanechi et al. (2016) and Shanechi et al. (2017).

#### 2.1.2. Time-frequency and time-scale features

Motor BCI transducers generally characterize analog population signal recordings via the temporal evolution of their frequency content [i.e., LFP (Aggarwal et al., 2013; Flint et al., 2013), ECoG (Chin et al., 2007; Yanagisawa et al., 2012; Wang et al., 2013a), and EEG (Pfurtscheller et al., 2000; Trejo et al., 2006; Hortal et al., 2015)].

The Fourier Transform is commonly used to disclose signals' frequency content. It permits us to decompose a time-domain signal *x*(*t*) ∈ ℝ onto a basis of complex exponentials of frequency *f*_*r*_ ∈ ℝ, yielding a frequency-domain complex signal $s\left(f_{r}\right)=\int_{-\infty }^{+\infty }x\left(t\right)e^{-\text{i}2\pi f_{r}t}dt$. Each frequency-specific component *s*(*f*_*r*_) is characterized by its phase ϕ(*f*_*r*_) = *arg*(*s*(*f*_*r*_)) and its amplitude |*s*(*f*_*r*_)|; that is, $s\left(f_{r}\right)=|s\left(f_{r}\right)|e^{i\phi \left(f_{r}\right)}$.

**Figure 4:**
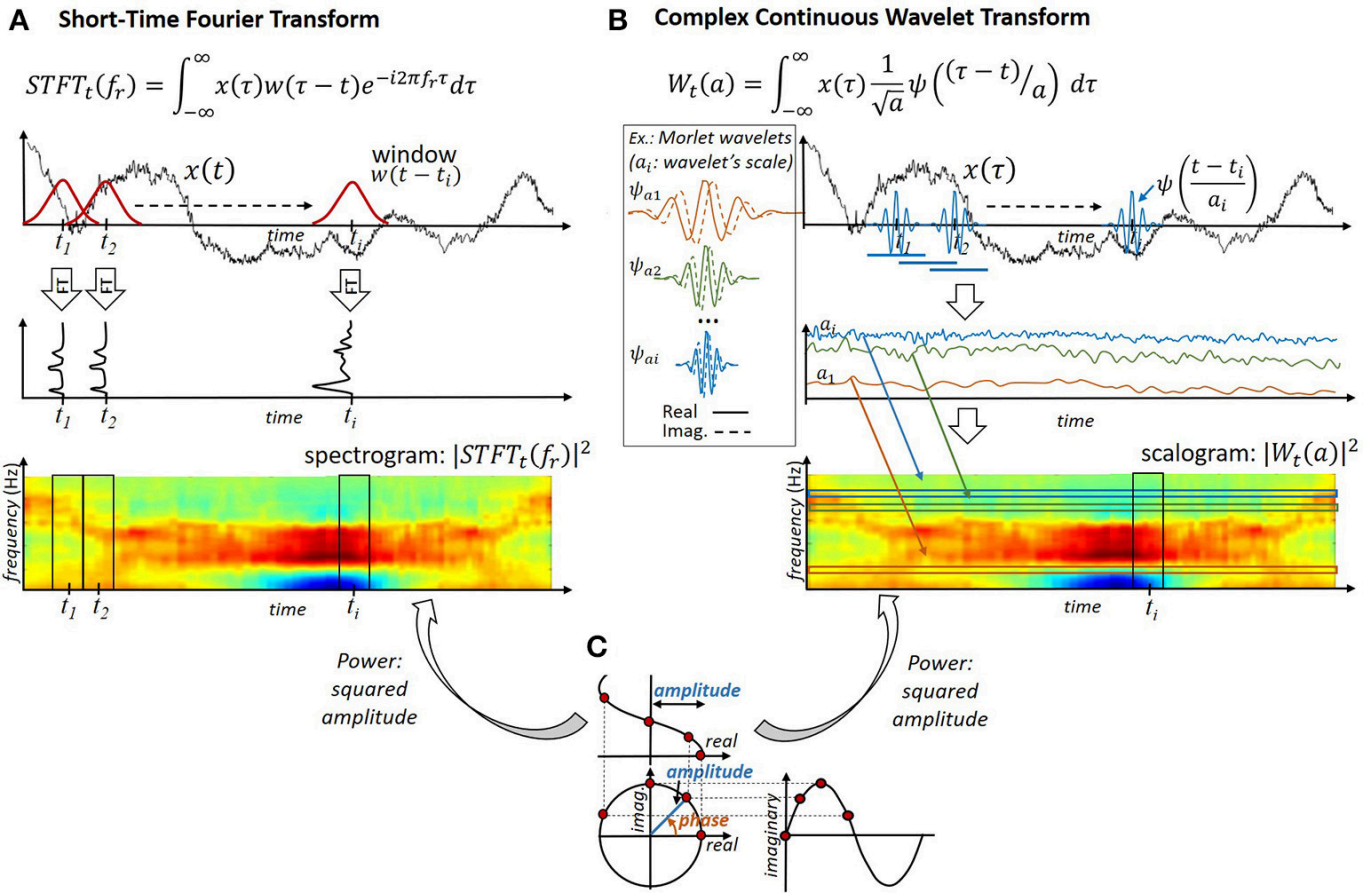
Extraction of phase, amplitude and power features from neural population recordings. **(A)** Short-Time-Fourier-Transform. The neural signal is windowed before application of the Fourier Transform. **(B)** Complex Continuous Wavelet Transform. The neural signal is convolved with complex wavelets of different scales. **(C)** Extraction of phase-, amplitude- and power features from a complex signal.

While the temporal variations of the neural signals' frequency content are expected to carry informations on the user's intents, the Fourier Transform does not permit us to readily describe these variations. Although the temporal information associated with a signal is contained in the phase ϕ(*f*_*r*_) = *arg*(*s*(*f*_*r*_)) of each Fourier component, it is not readily interpretable. Alternative approaches are thus used to describe the temporal evolution of a neural signals' spectral content. They generally consist of projecting, at different instants, the signal of interest onto real or complex oscillating components of different frequencies (e.g., a wavelet, a windowed complex exponential, etc.). As stated by the Eisenberg-Gabor uncertainty principle (Mallat, 2008), a perfect characterization of the signal frequency content at each instant is impossible. Therefore, these methods exhibit different time and frequency resolutions. Motor BCIs frequently exploit amplitude- and/or phase-based features extracted from the resulting real or complex time-frequency representations (Figure 4C).

**Amplitude-based features:** Features extracted from the amplitude of frequency-specific signals have been utilized in offline motor BCI studies (Chin et al., 2007; Wang et al., 2013a; Eliseyev and Aksenova, 2014) or online motor BCI systems (Yanagisawa et al., 2012). The amplitude or instantaneous power of frequency-specific components *s*_*t*_(*f*_*r*_)—that is, their squared amplitude $|s_{t}\left(f_{r}\right){|}^{2}$—are typically considered (Figure 4C). The utilization of the instantaneous value of these features measured at specific time moments has been reported in several motor BCIs, possibly after application of logarithm transform (Eliseyev and Aksenova, 2014). A second reported strategy consists of computing specific statistics associated with these features over a temporal window before feeding them to the decoder (Ball et al., 2009). The average is the statistic that is most frequently used to characterize amplitude-based features. However, the use of alternative statistics, such as the signal variance or other higher-order statistics, has also been investigated in motor BCI studies (Mahmoudi and Erfanian, 2002; Kevric and Subasi, 2017).

**Phase-based features:** Phase-based features (Figure 4C) have been exploited in several offline or online motor BCI studies. The phase information associated with each one of the channels of ECoG arrays has, for example, been used to reconstruct 1D trajectories in Hammer et al. (2013). A second class of phase features is based on the phase difference between signals, which permits us to characterize the coupling between channels. The Phase-Locking-Value (PLV) is defined as the average of the instantaneous phase difference in a temporal windows (Wei et al., 2007). The extraction of PLV features from EEG signals has been reported for the classification of motor imaginary tasks in several BCI studies (Gysels and Celka, 2004; Wang et al., 2006; Wei et al., 2007; Pourbakhtiar et al., 2013). In addition, the relevance of alternative phase-based features, such as the instantaneous or mean phase difference between two channels, has been investigated in the case of EEG signal in Hamner et al. (2011).

##### 2.1.2.1. Time-frequency representations

Amplitude and/or phase features are inferred from time-frequency representations of the neural signals. Different strategies have been implemented in motor BCIs' transducers to estimate the time-varying spectral content of the users' neural signals.

**Short-Time-Fourier-Transform:** The utilization of the Short-Time-Fourier-Transform (STFT)—that is, the computation of the Fourier Transform of temporally windowed neural signals (Figure 4A)—has been reported for the extraction of time-frequency features from ECoG (Chin et al., 2007; Yanagisawa et al., 2012) or EEG signals (Herman et al., 2005) in offline or online motor BCI studies. The amplitude (power) (Chin et al., 2007; Yanagisawa et al., 2012) and phase (Hammer et al., 2013) of the resulting time-frequency representation *s*_*t*_(*f*_*r*_) ∈ ℂ have been exploited in motor BCI studies. Given that the length of the window used to filter the signals is identical at all frequencies, the temporal and spectral resolution of the STFT is similar for all frequencies.

**Filter banks**: A filter bank is a set of band-pass filters. The STFT can, for example, be interpreted as a complex filter bank. Real-valued filter banks are also frequently applied to neural signals to extract signal components whose frequency content is included in a set of predefined frequency bands (Brodu et al., 2011). Most BCI studies exploit filter banks based on the Butterworth filter (e.g., Shin et al., 2012), which represent a trade-off between distortion in the frequency and time domains. For example, it has been used to extract time-frequency features from EEG (Bashashati et al., 2015) or ECoG signals (Nakanishi et al., 2013). In addition, the instantaneous value of ECoG band-pass filtered signals were utilized for trajectory reconstruction in Nakanishi et al. (2013). Filter banks were used to extract EEG power features in Bashashati et al. (2015). The instantaneous value of low-pass filtered neural signals has more specifically been reported to be tuned to upper-limb kinematic parameters and is regularly exploited in ECoG- (Schalk et al., 2007; Pistohl et al., 2008; Ball et al., 2009; Kellis et al., 2012; Milekovic et al., 2012; Wang et al., 2012; Hotson et al., 2014; Hammer et al., 2016), EEG- (Bradberry et al., 2010; Ofner and Müller-Putz, 2012) and LFP-based motor BCIs (Perge et al., 2014). Low-pass filtering is frequently performed by means of a Butterworth (Bradberry et al., 2010; Hammer et al., 2016), Savitzky-Golay (Pistohl et al., 2008; Ball et al., 2009; Kellis et al., 2012; Milekovic et al., 2012) or Moving Average (Wang et al., 2012; Hotson et al., 2014) filter. These temporal features are often combined with other time-frequency or time-scale features (Schalk et al., 2007; Wang et al., 2011).

**Wavelet transform**: Another approach reported in EEG- (Lemm et al., 2004; Bhattacharyya et al., 2011; Bashashati et al., 2015), ECoG- (Chao et al., 2010; Bhattacharyya et al., 2011; Shimoda et al., 2012; Eliseyev and Aksenova, 2014) and LFP-based (Bouton et al., 2016) motor BCI studies consists of applying a Wavelet Transform to compute a time-frequency representation of neural signals. The Wavelet Transform permits us to decompose signals onto real or complex signals (“wavelets,” see Figure 4B). Similarly to the STFT, it can be interpreted as a special case of filter banks. The squared amplitude of a wavelet transform is referred to as a “scalogram,” and the corresponding features are “time-scale” features. Complex wavelets permit us to decouple the signal's phase and amplitude, and thus give access to both characteristics independently (Torrence and Compo, 1998). In contrast, real wavelets return real time-frequency signals, which do not permit us to readily separate the amplitude and phase of the oscillating signals composing the analyzed signal (Torrence and Compo, 1998). The use of different wavelets has been investigated by, for example, Daubechies (Bhattacharyya et al., 2011; Bouton et al., 2016), Meyer (Eliseyev et al., 2012), Haar (Kousarrizi et al., 2009) or real (Chao et al., 2010; Bashashati et al., 2015), and complex (Lemm et al., 2004; Eliseyev and Aksenova, 2014) Morlet wavelets. More specifically, the relevance of different real and complex wavelets has been compared for kinematic decoding from ECoG signals in Eliseyev et al. (2012). Wavelet-based extraction of the instantaneous power (Chao et al., 2010; Shimoda et al., 2012) or amplitude (Eliseyev et al., 2012; Eliseyev and Aksenova, 2014) at specific instants has been reported for offline trajectory reconstruction from ECoG signals. Wavelet decomposition has also been embedded into the transducer of a BCI system, which permitted a quadriplegic user implanted with an intracortical array to control his own fingers (Bouton et al., 2016). In contrast with the STFT, the temporal resolution of the wavelet transform depends on the considered frequency because the duration of a wavelet depends on its scale.

**Hilbert transform**: Time-frequency features have been extracted by applying the Hilbert transform on band-pass filtered signals in several ECoG- (Hotson et al., 2016) or EEG-based (Gysels and Celka, 2004; Wang et al., 2006; Wei et al., 2007; Pourbakhtiar et al., 2013) BCI studies. The Hilbert transform permits us to compute the analytical signal *x*_*a*_(*t*) = *x*(*t*) + *jH*_*x*_(*t*) ∈ ℂ that is associated with a signal *x*(*t*), where *H*_*x*_(*t*) is obtained by convolving *x*(*t*) with $\frac{1}{\pi t}$. The instantaneous phase and amplitude of a signal are defined as the argument and modulus of the corresponding analytical signal. The amplitude and phase characteristics extracted by means of the Wavelet and Hilbert transforms have been found to be highly similar when specific wavelets and bandpass filters were utilized (Le Van Quyen et al., 2001; Bruns, 2004). Hilbert-based time-frequency representations have been used to estimate the high-gamma power of ECoG signals utilized by users to control prosthetic fingers in Hotson et al. (2016). Phase features have additionally been extracted from Hilbert-transformed EEG signals in several offline mental-task studies (Gysels and Celka, 2004; Wang et al., 2006; Wei et al., 2007; Pourbakhtiar et al., 2013).

**Empirical mode decomposition**: Empirical Mode Decomposition has been used in several EEG-based BCI studies (Park et al., 2013; Gaur et al., 2015, 2016; Kevric and Subasi, 2017). It relies on an iterative process to decompose a signal into several oscillating components, referred to as intrinsic mode functions (in particular, components which exhibit with similar numbers of extrema and zero-crossings). Contrary to the Fourier or Wavelet transforms, the shape of these modes are data-dependent, and are thus liable to adapt to the specificities of the signals. Empirical mode decomposition features have been reported to outperform wavelet-based features for EEG motor imagery classification (Abdalsalam et al., 2018). The computational complexity of EMD algorithms has additionally been shown to be similar to the one of the Fast Fourier Transform (Wang et al., 2014). Some of the drawbacks which may impair the efficiency of the EMD include border effects and difficulties to select the stopping criterion used to extract the intrinsic mode functions (Niang et al., 2010).

**Non-parametric spectrum estimation**: The previously enumerated methods are specifically designed to describe the temporal evolution of a signal's spectral content. An alternative strategy that is frequently utilized in BCI studies consists in applying generic power spectrum estimation methods to signals extracted via a sliding window. Some examples of the non-parametric methods used for power spectrum estimation in BCI studies include the periodogram (Brodu et al., 2011), Welch's periodogram (Millan et al., 2002; Cincotti et al., 2003) or multitaper analysis (Ball et al., 2009; Hasan and Gan, 2011). Both Welch's periodogram and multitaper analysis (Thomson, 1982) rely on the averaging of multiple spectra to reduce the variance of the corresponding spectrum estimate.

**Parametric spectrum estimation**: Finally, parametric spectrum estimation is a popular approach for the characterization of both ECoG (Leuthardt et al., 2004, 2011; Lal et al., 2005; Hill et al., 2006; Felton et al., 2007; Schalk et al., 2007, 2008; Blakely et al., 2009; Ashmore et al., 2012; Wang et al., 2012; Wang W. et al., 2013; Fifer et al., 2014) and EEG signals (Schlögl et al., 2005; Argunşah and Çetin, 2010) in online (Leuthardt et al., 2004, 2011; Felton et al., 2007; Blakely et al., 2009; Fifer et al., 2014) or offline (Schlögl et al., 2005; Hill et al., 2006; Argunşah and Çetin, 2010) motor BCI studies. The Auto-Regressive (AR) coefficients of the neural signals can be estimated via the Yule-Walker (Herman et al., 2008) or Burg method (Ashmore et al., 2012; Fifer et al., 2014). Spectrum estimation is then readily inferred from the AR parameters (Stoica et al., 2005). Although parametric estimation is computationally efficient, it relies either on a predefined and potentially suboptimal model order (Ashmore et al., 2012; Fifer et al., 2014) or on a model order selected after a possibly time-consuming optimization process (McFarland and Wolpaw, 2008). When it is based on Burg AR parameters, it is referred to as a maximum-entropy spectral estimation. Maximum-entropy spectral estimation has been performed in several offline ECoG studies (Anderson et al., 2012; Bundy et al., 2016; Spüler et al., 2016) and in an online motor EEG-based BCI system (Bundy et al., 2017).

##### 2.1.2.2. Integrating spatial information: time-frequency-space features

Most motor BCI transducers independently consider the signals provided by multi-channel arrays. Time-frequency-space features are thus obtained by concatenating the time-frequency features extracted for each channel.

More sophisticated strategies for the integration of spatial information into time-frequency features have been reported for both EEG- (Onose et al., 2012; Vidaurre et al., 2016) and ECoG-fed (Marathe and Taylor, 2013; Kapeller et al., 2015) motor BCI transducers. These strategies generally rely on Common Spatial Pattern (CSP) filters (Pfurtscheller and Neuper, 2001), which construct class-discriminative virtual channels under the criterion that their variance ratio is maximized between the two considered classes (Blankertz et al., 2008). The development of CSP variants is a particularly active research field. The Filter-Bank CSP (FBCSP) (Ang et al., 2012), whose computational efficiency has been reported in Aghaei et al. (2016), exploits a bank of bandpass filters to obtain neural signal rhythms in different frequency bands. The spatial features are then extracted by applying a separate CSP on each frequency. Some examples of the many CSP variants which have also been considered for motor imagery classification include common spatio-spectral patterns (Lemm et al., 2005), common sparse spectral spatial patterns (Dornhege et al., 2006), SPECtrally-weighted CSPs (Tomioka et al., 2006), iterative spatio-spectral patterns learning (Wu et al., 2008), sub-band CSPs (Novi et al., 2007), optimal spatiospectral filter networks (Zhang et al., 2011), filter bank CSPs (Ang et al., 2012), discriminative FBCSPs (Thomas et al., 2009), Bayesian spatio spectral filter optimization (Suk and Lee, 2013), discriminative filter-bank CSPs (Higashi and Tanaka, 2013), sparse filter band CSPs (Zhang et al., 2015) and bilinear separable common spatio-spectral patterns (Aghaei et al., 2016).

##### 2.1.2.3. Relevance of time-frequency features

**Time-frequency representations**: Comparative studies have investigated the relevance of different time-frequency features (e.g., features extracted by means of the STFT, wavelets, parametric spectrum estimation, etc.) for the classification of EEG signals elicited by motor imageries (Herman et al., 2008; Brodu et al., 2011). Parametric approaches are known to be particularly accurate when the considered signals can be satisfyingly represented by the chosen parametric model (Stoica et al., 2005). Non-parametric methods, e.g., non-parametric spectrum estimates, on the other hand, are theoretically more relevant when parametric models fail to closely approximate the signals of interest (Stoica et al., 2005). The results reported in comparative studies reflect this variable relevance of parametric and non-parametric approaches. The periodogram and parametric power estimation approaches are able to extract features associated with the best classification accuracy in Herman et al. (2008), whereas Morlet wavelet transforms have been found to surpass alternative methods (e.g., parametric power estimation) in Brodu et al. (2011). Similarly, wavelet-based features bettered AR-based features for non-motor imagery classification in Cabrera et al. (2010). These results seem to confirm that the relevance of different time-frequency and time-scale features partially depends on the datasets at hand.

**Amplitude and phase features**: Most motor BCI systems rely on amplitude features and they do not exploit the phase information of the neural signals. Nevertheless, phase-based neural encoding of information has been disclosed in several studies (Krusienski et al., 2011) and the interest of phase-based features has been suggested in offline and online motor BCI experiments. These have been shown to outperform amplitude features for 1D kinematic offline reconstruction from ECoG signals (Hammer et al., 2013) and they have permitted users to control a 3-class virtual effector in Brunner et al. (2006). However, the advantages of phase-related features to motor BCIs remains unclear. For example, in Krusienski et al. (2012) EEG phase and coherence features did not lead to an improved motor imagery classification accuracy when compared to Fourier features.

#### 2.1.3. Decoder-embedded feature extraction: end-to-end transducers

While feature extraction is a prerequisite for most motor BCI decoding algorithms, end-to-end learning—that is, learning from row data without any prior feature extraction—has recently been reported in several offline motor BCI studies (Wang Z. et al., 2013; Nurse et al., 2015b, 2016; Schirrmeister et al., 2017). In these studies, raw or preprocessed neural signals are directly fed to decoders. These models then learn how to both extract and decode useful neural signal characteristics during model identification. End-to-end learning has been investigated for movement classification (Nurse et al., 2015b, 2016; Schirrmeister et al., 2017) and trajectory prediction (Wang Z. et al., 2013) from EEG (Nurse et al., 2015b, 2016; Schirrmeister et al., 2017) and ECoG neural signals (Wang Z. et al., 2013) acquired either during motor imagery tasks or movement execution. These models generally rely on deep learning decoders, such as multi-layer perceptrons (Nurse et al., 2015b) or convolutional neural networks and their variants (Wang Z. et al., 2013; Nurse et al., 2016; Schirrmeister et al., 2017).

End-to-end learning exhibits several advantages; for example, it can be implemented with minimal preprocessing procedures [e.g., centering (Wang Z. et al., 2013; Nurse et al., 2015b; Schirrmeister et al., 2017), scaling (Wang Z. et al., 2013; Schirrmeister et al., 2017), outlier removal (Nurse et al., 2016), or band pass filtering (Yuksel and Olmez, 2015; Sturm et al., 2016; Tang et al., 2017)]. It additionally holds the promise of highly accurate decoding because of the joint optimization of feature extraction and decoding. While statistically significant performance improvements have been reported when comparing end-to-end models with approaches combining CSP-based feature extraction with generic classifiers (Yuksel and Olmez, 2015; Lu et al., 2017; Tang et al., 2017), end-to-end models have not yet clearly outperformed state of the art methods (Nurse et al., 2015b, 2016; Schirrmeister et al., 2017). Some of the difficulties that may impair the efficiency of end-to-end approaches include difficulties to fit end-to-end models, such as to gather enough data, and/or properly regularize the models so as to avoid overfitting.

#### 2.1.4. Other features

The use of alternative features such as fractal dimension, entropy measures or temporal sequence modeling has been proposed in motor imagery-based EEG-driven BCI studies (Boostani and Moradi, 2004; Coyle et al., 2005; Boostani et al., 2007; Zhang et al., 2008; Vidaurre et al., 2009). The efficient offline classification of EEG motor imageries has been achieved by exploiting the covariance matrices associated with each trial, and more specifically the Riemannian distance between these covariance matrices (Barachant et al., 2010, 2012). Amplitude coupling between a pair of channels has also been reported in motor BCI studies (Wei et al., 2006, 2007; Krusienski et al., 2012).

### 2.2. Features for effector control

Both discrete and continuous dependent variables can be estimated from neural signals to control prosthesis and orthosis movements.

Continuous variables traditionally consist of position and/or velocity of the effector's endpoint, such as the wrist kinematic parameters in the case of an upper-limb orthosis (Li, 2014), or of the angular characteristics of effector joints (Ajiboye et al., 2012). Wrist speed and acceleration (Hammer et al., 2013, 2016), force profile (Carmena et al., 2003; Chen C. et al., 2014), and muscular activity (Carmena et al., 2003; Koike et al., 2006; Choi et al., 2009; Shin et al., 2012) have been reconstructed in offline preliminary studies. The principal components of the effector's position or velocity have also been estimated from neural signals in offline studies (Acharya et al., 2010; Wong et al., 2013; Hotson et al., 2014).

Discrete variables in particular include the direction of the effector's movement (Bhattacharyya et al., 2015; Hortal et al., 2015), the finger of interest (Hotson et al., 2016) or the open/closed state in the case of hand prostheses or orthoses (Pfurtscheller et al., 2000). Binary dependent variables are also regularly used to characterize the state of the user during asynchronous decoding; that is, an Intentional Control (IC) or Non-Control (NC) state (Mason and Birch, 2000; Müller-Putz et al., 2010).

### 2.3. Dimensionality reduction

High dimensional and/or correlated features are liable to disrupt decoder training. They may, for example, result in ill-poised problems, in computational loads incompatible with real time requirements, or in an important user mental load caused by the acquisition of a large training dataset.

The reduction of the dimension of neural feature representations is mainly performed in offline or online motor BCI studies by means of projection methods, such as the principal component analysis and its variants (Devulapalli, 1996; Wu et al., 2003b; Kim S.-P. et al., 2006; Aggarwal et al., 2008; Ke and Li, 2009; Wang W. et al., 2009; Argunşah and Çetin, 2010; Suk and Lee, 2010; Bhattacharyya et al., 2011; Kao et al., 2013, 2017) or by means of feature selection methods, such as stepwise forward (Brunner et al., 2007; Liang and Bougrain, 2012; Wang et al., 2012; Hotson et al., 2014) or forward-backward (McFarland et al., 2010) selection procedures, LASSO-based sparse modeling methods (Least Absolute Shrinkage and Selection Operator) (Fazli et al., 2011; Kelly et al., 2012; Wang et al., 2015), so-called filter methods (Schalk et al., 2007; Spüler et al., 2016), genetic algorithms (Flotzinger et al., 1994; Graimann et al., 2004; Wei et al., 2006; Boostani et al., 2007; Fatourechi et al., 2007; Wei and Tu, 2008) or alternative approaches such as distinctive sensitive learning vector quantization (Flotzinger et al., 1994).

The optimal feature dimension depends on the complexity of the neural control task (e.g., number of degrees of freedom), on the number of parameters of the decoder, or on the associated identification approaches. Consequently, the feature dimension is generally treated as a hyperparameter that is not predefined but optimized for each particular application during the dimensionality reduction process.

## 3. Data-driven decoders

Feature extraction is followed by the application of a decoder which aims at translating features into estimates of the user's movement intention.

Let *x*^*t*^ ∈ ℝ^*m*^ be an independent, input variable and *y*^*t*^ ∈ ℝ^*n*^ or *y*^*t*^ ∈ ℤ denote a dependent, output variable. Let $\hat{f}$ be an estimate of the unknown model *f* such that *y*^*t*^ ≈ *f*(*x*^*t*^). When motor BCIs rely on the decoding of continuous variables *y*^*t*^ ∈ ℝ^*n*^ (Hochberg et al., 2012; Collinger et al., 2013; Wodlinger et al., 2015), the corresponding $\hat{f}:{\mathbb{R}}^{m}\to {\mathbb{R}}^{n}$ is referred to as continuous decoder (a regression model for example). These continuous decoders are typically used to build kinematic decoders. In the case of discrete dependent variables, a discrete decoder $\hat{f}:{\mathbb{R}}^{m}\to \mathbb{Z}$ (classifier) is applied on neural features (Tsui et al., 2007; Yanagisawa et al., 2012; Hotson et al., 2016). Discrete decoders are generally used for the task of mental-task-based effector control.

The adaptation of user-specific decoders is carried out in the majority of motor BCIs. Machine learning methods are used to build a relevant decoder $\hat{f}$ to model the dependence between neural features *x*^*t*^ and user intentions *y*^*t*^. This model is designed to maximize its decoding performance.

### 3.1. Performance indicators

The performance of a decoder is generally measured by means of one or several indicators. These indicators are used to choose the decoder structure, optimize its hyperparameters, and monitor user training.

#### 3.1.1. Open-loop performance

While the ultimate goal of a motor BCI system is the ability of the patient to control an orthosis or prosthesis device, model structure and hyperparameters are generally optimized on open-loop data. Several different metrics have been used to assess the open-loop performance of discrete and continuous decoders to be embedded into motor BCI transducers.

**Discrete decoders**: Many performance indicators have been proposed to assess the performance of discrete decoders (Mason et al., 2006). Several indicators have been derived from the confusion matrix (e.g., the classification accuracy or classification error) and they are regularly used in motor BCI studies (Velliste et al., 2014; Bundy et al., 2016). While these metrics are relevant measures of the global classification quality when classes are well balanced (Mason et al., 2006; Thomas et al., 2013), alternative indicators, such as the Kappa coefficient, Nykopp's mutual information (Mason et al., 2006) or the Area Under the ROC (Receiver Operating Characteristic) Curve can be profitably used in the case of unbalanced classes. Although the simultaneous computation of the true positive rate and false positive rate also provides useful insights on classification performance, comparison between decoders is eased by the utilization of a single metric. Finally, the information transfer rate is regularly utilized to facilitate the comparison between decoders trained on different classification tasks, such as binary or multi-class tasks. This enables us to combine the decoding task difficulty with the corresponding decoder performance (Schögl et al., 2007). Guidelines for the choice of discrete performance indicators in function of class balance and decoder bias are, for example, available in Thomas et al. (2013).

**Continuous decoders**: The accuracy of continuous variable estimates (e.g., the reconstruction of 3D trajectory) is typically assessed via the Pearson Correlation Coefficient (PCC) and/or the Root-Mean-Squared Error (RMSE) (Spuler et al., 2015), such as in Velliste et al. (2014) and Bundy et al. (2016). The PCC reflects the amount of linear dependence between the observed *y* and estimated ŷ variables. The RMSE measures the ℓ^2^-error between both variables. The mean absolute error, which indicates the ℓ^1^-error between the vectors of observations, has sometimes been used to assess the fidelity of trajectory reconstruction (Eliseyev and Aksenova, 2014) because it is less sensitive to outliers than the RMSE (Hyndman and Koehler, 2006). The coefficient of determination associated with a regression model is also frequently reported (Wang et al., 2007; Marathe and Taylor, 2013). Meanwhile, alternative indicators generally focus on trajectory delay and smoothness, which are liable to impact the user control performance (Marathe and Taylor, 2015). The interpretation of the level of such indicators (i.e., the corresponding ability of a motor BCI user to execute daily life movements) is not straightforward. PCCs superior to 0.75 or 0.5 have, for example, been shown to be required for patients to efficiently execute reaching movements in the case of position and velocity decoding, respectively (Marathe and Taylor, 2011).

#### 3.1.2. Closed-loop performance

While open-loop indicators provide the necessary tools for the analysis of open-loop data, specific performance indicators have been considered for closed-loop BCIs. In particular, kinematic BCIs are often evaluated by means of center-out reaching tasks. Cursor speed, trajectory error, path efficiency, success rate, hold-on-target error rate are some of the indicators regularly reported in closed-loop motor BCI studies (Collinger et al., 2013; Gowda et al., 2014; Wodlinger et al., 2015). It has additionally been suggested to utilize the information transfer rate (Tehovnik et al., 2013) to compare the performance of decoders for reach-out tasks (Baranauskas, 2014).

### 3.2. Discrete decoding: classifiers

**Figure 5:**
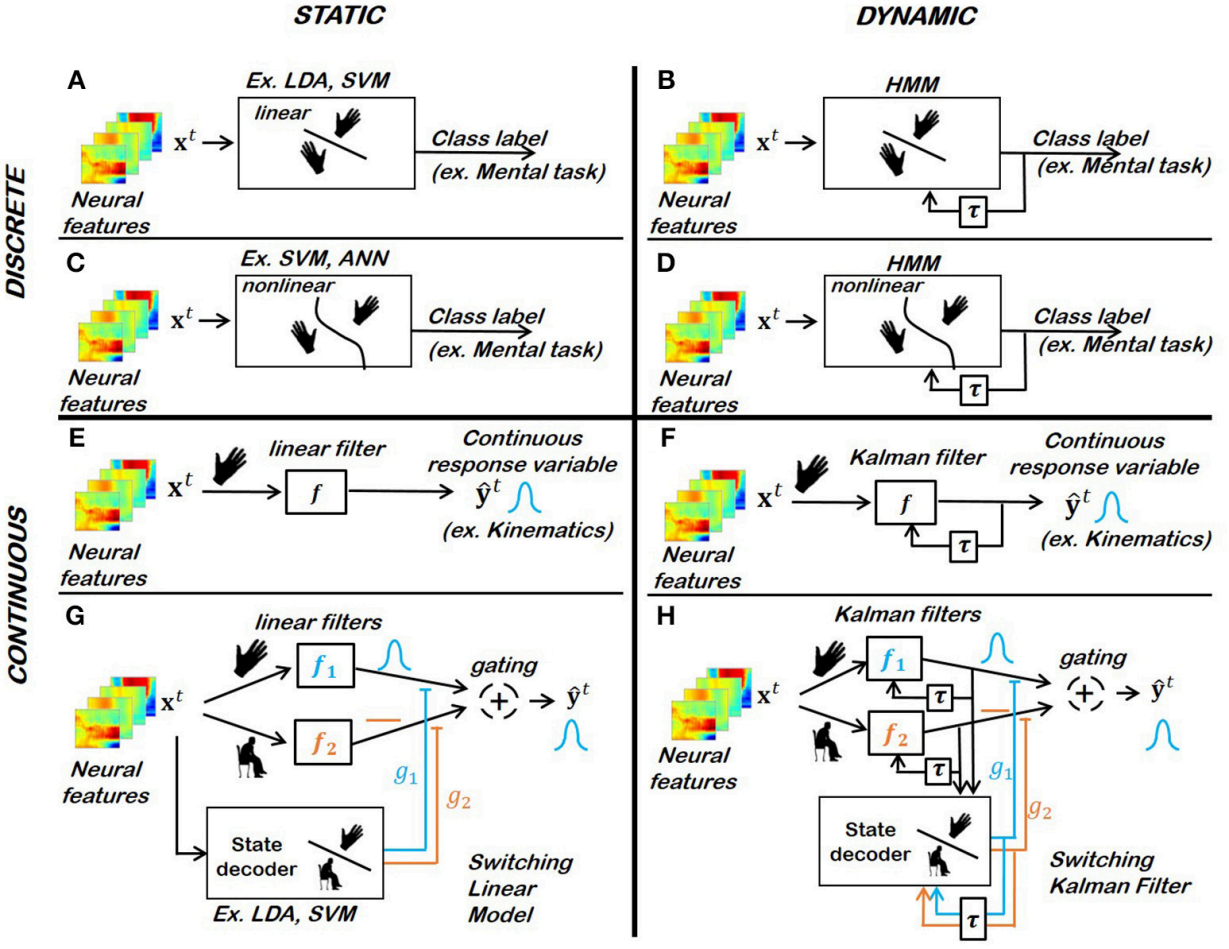
Popular motor BCI decoders for biomimetic kinematic and mental-task decoding strategies. **(A)** Linear static classifier for discrete response variables (Yanagisawa et al., 2012; Hotson et al., 2016). **(B)** Linear dynamic classifier for discrete response variables (Fifer et al., 2014). **(C)** Non-linear static classifier for discrete response variables (Kayikcioglu and Aydemir, 2010; Bhattacharyya et al., 2011). **(D)** Non-linear dynamic classifier for discrete response variables. **(E)** Linear static filter for continuous response variables (Collinger et al., 2013; Wodlinger et al., 2015). **(F)** Linear dynamic filter for continuous response variable (Wu et al., 2002; Hochberg et al., 2012). **(G,H)** Switching static or dynamic decoders (Wu et al., 2003b; Bundy et al., 2016).

A discrete-valued dependent variable is usually referred to as “class label.” The associated observation *x*^*t*^ is said to belong to the “class” identified by its label. The classification of neural patterns [e.g., the discrimination between different mental tasks such as motor imageries or idle states (Fifer et al., 2014)] has been used as the basis of several EEG- and ECoG-driven motor BCIs (Tsui et al., 2007; Yanagisawa et al., 2012; Hortal et al., 2015; Hotson et al., 2016). The performance of various classifiers has been investigated, either in offline preliminary studies or in online preclinical or clinical studies. The classifier structures detailed in this section are summarized in Figure 5.

#### 3.2.1. Generative and discriminative classifiers

Both generative and discriminative classifiers have been used in EEG- (Chae et al., 2012; Hortal et al., 2015), ECoG- (Yanagisawa et al., 2012; Fifer et al., 2014; Kapeller et al., 2015), and LFP-based (Aggarwal et al., 2013) offline or online motor BCI studies.

##### 3.2.1.1. Generative classifiers

The first category of classifiers, namely generative classifiers, focuses on the neural feature distribution within each class. The use of generative classifiers has been reported in both EEG- (Pfurtscheller et al., 2000; Tsui et al., 2007; Chae et al., 2012; Vidaurre et al., 2016) and ECoG-based online motor BCI studies (Fifer et al., 2014; Kapeller et al., 2015; Hotson et al., 2016). The relevance of generative classifiers has additionally been investigated in offline EEG- (Chiappa and Bengio, 2003; Hasan and Gan, 2009; Bhattacharyya et al., 2011), ECoG- (Wang et al., 2016) and MUA/SUA-based (Hatsopoulos et al., 2004) preliminary studies; for example, for the classification of real movements (Hatsopoulos et al., 2004; Wang et al., 2016) or of mental tasks (Chiappa and Bengio, 2003; Hasan and Gan, 2009; Bhattacharyya et al., 2011).

Generative classifiers model the joint probability *P*(*x*^*t*^, *y*^*t*^ = *i*) for each considered class *i* (Ng and Jordan, 2002). Once the joint probability has been fitted for each class, the classification of a new observation sample *x*^*t*^ is performed by computing the posterior probability *P*(*y*^*t*^ = *i*|*x*^*t*^) with respect to each class (Ng and Jordan, 2002). The equiprobable hypersurface defined by *P*(*y*^*t*^ = *i*|*x*^*t*^) = *P*(*y*^*t*^ = *j*|*x*^*t*^) is referred to as “decision boundary.” Its characteristics (e.g., linearity or non-linearity) are not explicitly chosen but instead result from the distribution used to model data generation within each class. The majority of generative classifiers reported in motor BCIs or preliminary studies relied on multivariate Gaussian distributions (e.g., Lemm et al., 2004; Bhattacharyya et al., 2011; Aggarwal et al., 2013; Do et al., 2013b; Wang et al., 2016) or Gaussian mixtures models (Chiappa and Bengio, 2003; Hasan and Gan, 2009). By contrast, the MUA/SUA firing rates of two non-human primates were advantageously modeled by Poisson distributions in Hatsopoulos et al. (2004). Finally, prior knowledge on the parameters of the considered distributions can be integrated via Bayesian learning strategies (Zhang et al., 2016).

While high-dimensional neural features are frequently extracted from neural signals in offline and online motor BCI studies (Kim et al., 2011; Bhattacharyya et al., 2015) (up to 285 and 630 features, respectively), fitting high-dimensional multivariate distributions is a delicate matter (Fan et al., 2011). To overcome this problem, the use of naive Bayes classifiers has been investigated by several teams (Hatsopoulos et al., 2004; Santhanam et al., 2006; Bhattacharyya et al., 2011; Chestek et al., 2013); for example, to classify eight wrist directions on the basis of the firing rate of 32 to 143 neurons in Hatsopoulos et al. (2004). Naive Bayes classifiers assume that features are independent conditionally to the class. Therefore, classifier training consists of fitting one univariate distribution per feature rather than one multivariate distribution for the full feature set. The performance of a Naive Bayes and of a Gaussian-based generative classifier was compared for left- and right-hand movement classification from more than 800 EEG features in Bhattacharyya et al. (2011). The naive Bayes classifier reportedly surpassed the generic Bayes classifier, both when the full EEG feature set was considered and when its dimensionality had been preliminary reduced to 91 by means of Principal Component Analysis.

##### 3.2.1.2. Discriminative classifiers

Discriminative classifiers explicitly model the class decision boundary. They have been utilized in both EEG- and ECoG-driven motor BCIs; for example, for EEG-based displacement of a robotic arm's endpoint (Hortal et al., 2015) or for ECoG-based control of a prosthetic arm (Yanagisawa et al., 2012). Their performance has also been assessed in offline preliminary studies; that is, for the classification of motor imageries (Schlögl et al., 2005; Hill et al., 2006; Chin et al., 2007).

Discriminative classifiers directly model the posterior class probability *P*(*y*^*t*^ = *i*|*x*^*t*^) (Bishop, 2006). This makes the use of discriminative classifiers particularly relevant when the distribution of neural features within each class cannot be approximated with classical distributions. Discriminative classifiers are particularly relevant when *x*^*t*^ is high-dimensional or includes redundant (correlated) neural features (Sutton and Mccallum, 2012) because non-discriminant features are not considered during model training.

Some of the discriminative classifiers whose use has been reported in offline or online motor BCI studies include Support-Vector-Machine classifiers (Lal et al., 2005; Schlögl et al., 2005; Hill et al., 2006; Sadeghian and Moradi, 2007; Bhattacharyya et al., 2011; Yanagisawa et al., 2012; Hortal et al., 2015), Logistic Regression (Tomioka et al., 2007; Chen W. et al., 2014; Bashashati et al., 2015; Bundy et al., 2016), k-Nearest Neighbors (Chin et al., 2007; Kayikcioglu and Aydemir, 2010) and Artificial Neural Networks (Haselsteiner and Pfurtscheller, 2000; Hatsopoulos et al., 2004; Navarro et al., 2005; Nakayama and Inagaki, 2006; Kumar et al., 2016; Nurse et al., 2016; Sturm et al., 2016; Schirrmeister et al., 2017; Tang et al., 2017).

##### 3.2.1.3. Generative vs. discriminative classifiers

The superiority of generative or discriminative classifiers for mental states classification generally depends on the problem at hand, particularly on the neural feature dimension, and they cannot be established *a priori*. Both types of classifiers are regularly utilized in offline and online motor BCI studies. For example, Linear Discriminant analysis and Support-Vector-Machine classifiers, both of which are widely popular within the BCI community (Nicolas-Alonso and Gomez-Gil, 2012; Bashashati et al., 2015) are a generative and a discriminative classifier, respectively. Generative and discriminative classifiers can be associated with linear or non-linear decision boundaries.

#### 3.2.2. Linear and non-linear classifiers

Both linear (Tsui et al., 2007; Fifer et al., 2014; Hortal et al., 2015; Kapeller et al., 2015; Hotson et al., 2016; Vidaurre et al., 2016) and non-linear classifiers have been exploited in motor BCI systems recently deployed during online experiments.

##### 3.2.2.1. Linear classifiers

Several different linear classifiers have been applied and tested for online or offline neural pattern classification for motor BCIs.

**Linear discriminant analysis**: Linear Discriminant analysis (LDA) classifiers are generative classifiers that are based on multivariate Gaussian distributions which covariance matrix is shared among classes. LDA classifiers have been embedded in several motor BCIs, such as in BCIs providing users with control over hand prostheses or orthoses (Pfurtscheller et al., 2000; Fifer et al., 2014; Hotson et al., 2016), lower-limb orthoses (Vidaurre et al., 2016) or humanoid robots (Kapeller et al., 2015). LDA has also been used for offline motor imagery classification in EEG (Bhattacharyya et al., 2011), and for the discrimination between motor states (e.g., idleness, movement, immobilization over a target after a reaching movement) estimation in LFP (Aggarwal et al., 2013) and MUA/SUA (Velliste et al., 2014) signals.

**Support-Vector-Machines**: Support-Vector-Machines (SVM) have frequently been applied in motor BCI studies (Schlögl et al., 2005; Yanagisawa et al., 2012; Hortal et al., 2015) The SVM's linear decision boundary is chosen so as to maximize its margin with the nearest training samples (Bishop, 2006). SVM-based classification has for example enabled users to control a prosthetic hand (Yanagisawa et al., 2012) and a robotic arm (Hortal et al., 2015). SVMs are also regularly used for offline motor imagery classification in ECoG (Lal et al., 2005; Hill et al., 2006; Demirer et al., 2009; Yanagisawa et al., 2012) and EEG (Schlögl et al., 2005; Sadeghian and Moradi, 2007; Bhattacharyya et al., 2011) signals. SVMs are attractive for neural signal decoding (Lotte et al., 2007) because of their good generalization abilities (Schlögl et al., 2005) and because of their robustness in high-dimensional settings (Friedman et al., 2001).

**Thresholded linear regression model**: The application of a threshold on the output of a linear regression model has been reported in motor BCI studies, such as for ECoG-driven asynchronous 2D cursor control (Williams et al., 2013). This classification approach has also been used for offline discrimination between active and idle states from ECoG signals (Eliseyev et al., 2011, 2012; Costecalde et al., 2017).

**Logistic regression**: Logistic Regression (LR) is a discriminant classifier that is based on generalized linear models, which extend linear models in that a non-linear link function *g* is applied on a linear combination of features (Bishop, 2006). In contrast with linear regression-based classifiers, LR considers a discrete dependent variable and assumes that *P*(*y*^*t*^|*x*^*t*^) follows a Bernoulli distribution. Several teams have investigated its relevance for the discrimination between motor imageries or actions from EEG (Tomioka et al., 2007; Gouy-Pailler et al., 2009; Bashashati et al., 2015) and ECoG signals (Chen W. et al., 2014; Bundy et al., 2016).

The respective performance of linear classifiers for motor BCI systems is still a matter of debate. LDA has been regularly used to provide users with neural control over prostheses, orthoses, and robotic devices, and it is particularly popular for EEG offline linear classification (Bashashati et al., 2015). However, no clear superiority of LDA decoding performance has been reported in offline comparative studies (Schlögl et al., 2005; Wang W. et al., 2009; Bashashati et al., 2015). In Bashashati et al. (2015), a LDA classifier was slightly but not significantly surpassed by a LR-based classifier for EEG decoding. In Wang B. et al. (2009), LDA and SVM performed similarly for both motor imagery and finger movement classification from EEG signals. In another comparative study (Schlögl et al., 2005), LDA was significantly outperformed by a SVM for 4-class motor imagery classification in EEG signals. By contrast, it performed better than a SVM when applied on low-dimensional EEG features in Bhattacharyya et al. (2011). Its comparatively low robustness in high dimensions was also illustrated in the same study (Bhattacharyya et al., 2011), as LDA performance diminished when the dimension of the EEG features had not been reduced beforehand (Bhattacharyya et al., 2011).

##### 3.2.2.2. Non-linear classifiers

Non-linear classifiers have mainly been applied in offline preliminary studies, such as to discriminate between several motor imageries (Schlögl et al., 2005; Bhattacharyya et al., 2011; Sturm et al., 2016; Schirrmeister et al., 2017), cognitive tasks (Nakayama and Inagaki, 2006) or real movements (Navarro et al., 2005; Nurse et al., 2016).

**Quadratic discriminant analysis**: Several teams have reported EEG mental task classification by means of Quadratic Discriminant analysis (QDA) classifiers; that is, Gaussian-based generative classifiers with class-specific covariance matrices (Schlögl et al., 2005; Bhattacharyya et al., 2011).

**Non-linear SVMs**: The use of non-linear SVM has been investigated for EEG feature classification in Bhattacharyya et al. (2011) and Bashashati et al. (2015). Non-linear SVMs were designed by means of non-linear kernels, typically Radial Basis Functions (RBF), in Bhattacharyya et al. (2011) and Bashashati et al. (2015).

**Artificial neural networks**: Artificial neural networks (ANNs) attempt to mimic information encoding in biological neuron networks (Bishop, 2006) by applying cascaded non-linear functions on weighted combinations of features, resulting in a highly non-linear model (Bishop, 2006). ANNs have been used for offline, non-linear classification of motor imageries from EEG signals (Haselsteiner and Pfurtscheller, 2000; Mahmoudi and Erfanian, 2002; Navarro et al., 2005; An et al., 2014; Ren and Wu, 2014; Nurse et al., 2015b; Sakhavi et al., 2015; Yuksel and Olmez, 2015; Kumar et al., 2016; Sturm et al., 2016; Lu et al., 2017; Schirrmeister et al., 2017; Tabar and Halici, 2017; Tang et al., 2017) or of real movements from EEG or ECoG signals (Navarro et al., 2005; Nurse et al., 2016). The flexibility of ANNs makes them attractive for the complex problem of neural signal modeling. Among ANNs, Deep Neural Networks (that is, ANNs which apply several layers rather than a single layer of functions to the input features), have recently gained much popularity in the machine learning community (LeCun et al., 2015). The use of Deep Neural Networks has for example been reported for motor imagery and real movement classification from EEG recordings (Nurse et al., 2016; Schirrmeister et al., 2017).

**k-nearest neighbors**: Finally, the use of the k-Nearest Neighbors (kNN) classifier has been investigated for offline detection of motor imageries (Schlögl et al., 2005; Kayikcioglu and Aydemir, 2010; Bhattacharyya et al., 2011) and/or actions (Mason and Birch, 2000; Wang W. et al., 2009) from EEG features, for real movement classification from ECoG data (Chin et al., 2007) and for target estimation from primate MUA/SUA signals (Ifft et al., 2013). In contrast with previously reported classifiers, the kNN classifier is not parametric. A new sample is assigned with the label which is the most represented among its *k* nearest training samples (Bishop, 2006). Thus, kNNs do not require a time-consuming training procedure to be completed. However, a high computational load can be associated with the application of the kNN, inasmuch as the latter necessitates computing the distance between a new sample and all training samples. This shortcoming may limit its applicability for motor BCIs, as online kNN-based classification may introduce a large delay into the system.

Mixed results have been reported in the comparative studies completed on non-linear classifiers (Wang B. et al., 2009; Kayikcioglu and Aydemir, 2010).

While ANNs exhibit a high capability of modeling non-linear relationships between neural signals and dependant variables, it has nevertheless been reported that they can suffer from a few shortcomings, namely: difficulties to select the optimal network architecture, to avoid overfitting (Kayikcioglu and Aydemir, 2010), and to interpret results. Consequently, it has been observed in comparative studies that the accuracy of ANN-based mental task classification is not systematically better than the one obtained with simple non-linear models (Garrett et al., 2003; Wang B. et al., 2009). In Kayikcioglu and Aydemir (2010), an ANN was outperformed by a non-linear SVM for different training dataset sizes. In Garrett et al. (2003), where non-linear SVMs and ANNs were compared for a 5-class discrimination task with EEG signals, the ANN was bettered by the SVM. Similar results were obtained on two EEG datasets in Wang B. et al. (2009).

Similar observations have been reported for kNN classifiers. In Wang B. et al. (2009), a kNN performed similarly to a SVM with a RBF kernel for the discrimination between EEG motor imageries, and was only slightly surpassed by the same SVM-based classifier for finger movement decoding from EEG signals. This satisfying performance was obtained with low-dimensional input features (respectively, of 2 and 14). In Kayikcioglu and Aydemir (2010), a similar comparison was drawn between a kNN, a RBF-based SVM and an ANN for 2-class classification in the context of EEG-based up-down neural control of a cursor. The kNN outperformed both the MLP and SVM for this specific classification task and its performance was best maintained when the researchers attempted to reduce the training dataset size. However, the comparison was performed in a setting particularly favorable to the kNN because the input features were only of dimension two. By contrast, in Bhattacharyya et al. (2011), the kNN was outperformed by a RBF-based SVM for two sizes of independent variable (namely, 871 and 91 features).

##### 3.2.2.3. Respective relevance of linear and non-linear models

The respective interest of non-linear and linear classifiers for motor BCIs is still unclear. First, most of the previously mentioned classifiers have not been used for online pattern classification. Additionally, offline comparisons have generally been completed for two or three classifiers only and the statistical significance of the results has seldom been established. However, a few studies have endeavored to assess the relative interest of linear and non-linear classifiers for offline discrimination between motor imageries or actions (Müller et al., 2003; Wang B. et al., 2009; Bhattacharyya et al., 2011; Bashashati et al., 2015) or between cognitive tasks (Garrett et al., 2003).

Linear models exhibited a lesser modeling ability in several offline motor studies (Wang B. et al., 2009; Bhattacharyya et al., 2011; Tang et al., 2017). A RBF-based SVM was found to outperform a linear SVM as well as the other linear classifiers for both motor imagery and finger movement classification in Bhattacharyya et al. (2011) and Wang B. et al. (2009). In Tang et al. (2017), a convolutional ANN bettered a linear SVM fed with generic neural features.

The superiority of non-linear classifiers has not, however, been systematically reported in offline motor BCI. For example, QDA did not outperform LDA in two comparative studies (Wang B. et al., 2009; Bhattacharyya et al., 2011). In Garrett et al. (2003), a LDA classifier was compared to non-linear SVMs and to an ANN for the classification of five mental tasks. The performance of the non-linear classifiers was found to be only slightly more efficient than the LDA's one for this EEG classification task. In Sturm et al. (2016), an ANN was bettered by a SVM fed with CSP-based features. The training pitfalls associated with non-linear models were illustrated in Schlögl et al. (2005), where a kNN was significantly outperformed by a linear SVM and by LDA for 4-class motor imagery discrimination from EEG signals. Consequently, Müller et al. (2003) advocated the use of linear methods except for some specific cases with “complex, large” datasets. Correspondingly, linear classifiers like LDA (Bashashati et al., 2015) are regularly chosen over non-linear models despite their lesser modeling ability. In particular, most recent clinical motor BCIs have relied on linear classifiers (e.g., LDA Tsui et al., 2007; Fifer et al., 2014; Kapeller et al., 2015; Hotson et al., 2016; Vidaurre et al., 2016 or SVM Hortal et al., 2015).

Despite some trends, the relevance of a linear or non-linear classifier ultimately depends on the problem at hand. In Bashashati et al. (2015), for example, the two top classifiers for self-paced data decoding were a linear and a non-linear classifier, namely a LR classifier and an ANN. Classifier performance is particularly related to the characteristics of the extracted neural features, for example to their type (Bashashati et al., 2015) or dimension (Bhattacharyya et al., 2011). In Bashashati et al. (2015), the classifiers' performance for synchronous data decoding was not similar when classifiers were fed with band-pass- or with wavelet-based features. In Bhattacharyya et al. (2011), differences in performance ranking were observed if classifiers were applied on a high-dimensional input variable or on the same variable after PCA-based dimensionality reduction. Similarly, a RBF SVM and a kNN were identified as the best classifiers for a task of motor imagery decoding in Wang B. et al. (2009) but LDA reportedly equalled a RBF-based SVM for finger decoding.

Finally, to the best of our knowledge, only limited comparative studies have been completed on ECoG data (Shenoy et al., 2008). The respective relevance of the above-mentioned classifiers thus remains to be ascertained for ECoG data.

#### 3.2.3. Static and sequential classifiers

The previously mentioned classifiers are static; that is they don't take into account possible dependencies between successive independent or dependent variables. This assumption is typically violated in motor BCI studies. More specifically, such dependencies are likely to occur during closed-loop BCI acquisition sessions, as users take the classifier's past outputs into account when determining their current movement intention.

A few teams have investigated the interest of taking into account the sequential nature of the neural features or of the class labels (Obermaier et al., 2001; Chiappa and Bengio, 2003; Argunşah and Çetin, 2010). One strategy regularly utilized in motor offline or online BCI studies, such as Kim et al. (2011), Flamary and Rakotomamonjy (2012), and Eliseyev et al. (2012), consists in extracting features from several time segments to build a temporal sequence of feature vectors. This sequence is then fed to a static classifier (Dietterich, 2002; Lotte et al., 2007). Another approach, namely the application of dynamic classifiers, has been reported for neural pattern classification in SUA/MUA (Darmanjian et al., 2003), EEG (Obermaier et al., 2001; Argunşah and Çetin, 2010), and ECoG-based (Onaran et al., 2011; Delgado Saa et al., 2016) BCI studies. Dynamic classifiers directly exploit time series temporal behavior (Lotte et al., 2007).

**Hidden Markov Models**: The dynamic classification of neural signals has been performed by means of Hidden Markov Models (HMMs) in EEG (Obermaier et al., 2001; Štastný and Sovka, 2007; Gouy-Pailler et al., 2009; Argunşah and Çetin, 2010), ECoG (Onaran et al., 2011) and SUA/MUA offline motor BCI studies (Darmanjian et al., 2003; Wissel et al., 2013), and in a few online motor BCIs (Fifer et al., 2014; Hotson et al., 2016).

HMMs consider a hidden state *z*^*t*^ ∈ ℤ, which is generated by a first order Markov process, such as *P*(*z*^*t*+1^ = *k*|*z*^1:*t*^) = *P*(*z*^*t*+1^ = *k*|*z*^*t*^) (Rabiner, 1989). The value of the observation *x*^*t*^ ∈ ℤ^*m*^ or *x*^*t*^ ∈ ℝ^*m*^ depends on the corresponding hidden state value *z*^*t*^ via the conditional probability *P*(*x*^*t*^|*z*^*t*^) (Rabiner, 1989). Bayes filtering provides efficient recursive algorithms to infer the most likely state label ẑ^*t*^ by combining prior knowledge about the previous hidden state *z*^*t*−1^ with the likelihood of the current observed features *x*^*t*^ (Rabiner, 1989).

One approach reported for neural signal HMM-based classification consists in associating one hidden state value *z*^*t*^ = *i* with each class label *y*^*t*^ = *i* (Kemere et al., 2008; Fifer et al., 2014; Hotson et al., 2016). This strategy has, for example, been used for offline target estimation from SUA/MUA signals in (Kemere et al., 2008). HMM-based classifiers have also applied for robust online state detection in several closed-loop motor BCIs (Fifer et al., 2014; Hotson et al., 2016; Kao et al., 2017). States were, for example, associated to NC and IC classes (Fifer et al., 2014; Hotson et al., 2016).

An alternative approach has been investigated in offline preliminary studies (Obermaier et al., 2001; Darmanjian et al., 2003; Argunşah and Çetin, 2010; Onaran et al., 2011; Wissel et al., 2013). One HMM was associated with each considered class and several states were thus used to model feature dynamic within each class. Classification was performed by feeding each HMM with a sequence of *N* consecutive observations and by computing the associated probability *P*(*x*^*t*−*N*+1:*t*^|*y*^*t*^ = *i*).

The sequence was assigned the class *i*, which maximized *P*(*x*^*t*−*N*+1:*t*^|*y*^*t*^ = *i*). HMMs have been used for offline modeling of the variations variations of neural features within No-Control and Intentional Control states in SUA/MUA (Darmanjian et al., 2003) and ECoG (Onaran et al., 2011), within finger movements in ECoG signals (Wissel et al., 2013), or within motor imageries in EEG signals (Obermaier et al., 2001; Argunşah and Çetin, 2010).

The use of HMM's variants has been proposed for the classification of EEG and ECoG mental tasks (Chiappa and Bengio, 2003; Awwad Shiekh Hasan and Gan, 2010; Hasan and Gan, 2011; Saa and Çetin, 2012; Saa and Çetin, 2013).

**Input-output hidden Markov models**: Input-Output Hidden Markov Models (IOHMMs), which integrate both the modeling of each hidden state's dynamic and of class succession, have been applied on EEG signals to discriminate between 3 mental tasks in Chiappa and Bengio (2003). In contrast with HMMs, IOHMMs are trained to distinguish between classes composed of several hidden states, and directly map input features to the non-stationary classes (Bengio and Frasconi, 1996).

**Conditional random fields**: Conditional Random Fields (CRFs) are discriminative undirected graphical models (Sutton and Mccallum, 2012), and linear-chain CRFs are more specifically the discriminative counterpart of HMMs (Sutton and Mccallum, 2012). CRFs have been used for EEG offline modeling and decoding (Awwad Shiekh Hasan and Gan, 2010; Hasan and Gan, 2011; Saa and Çetin, 2012; Saa and Çetin, 2013), and for finger movement detection in ECoG signals (Delgado Saa et al., 2016). While they have a better ability to model long-term time dependencies (Lafferty et al., 2001), their training is computationally expensive (Dietterich, 2002).

**Dynamic Bayesian models**: Dynamic Bayesian Models (DBNs) are probabilistic graphical models which permit to take into account the dependence between several random variables (Murphy, 2002). HMMs are a specific case of DBNs and are, therefore, less flexible than DBNs. The dynamic of EEG (Shenoy, 2005) and ECoG (Wang et al., 2012) signals has been exploited by means of DBN (Murphy, 2002) in offline studies.

**Recurrent neural networks**: Finally, the use of a time-dependent ANN was reported for EEG dynamical classification in Haselsteiner and Pfurtscheller (2000).

Most dynamic classifiers which were embedded in motor BCIs were generic HMMs with state-class correspondence (Fifer et al., 2014; Hotson et al., 2016; Kao et al., 2017). Although the respective performances of more complex dynamical classifiers has been investigated and compared in offline studies (Chiappa and Bengio, 2003; Saa and Çetin, 2012; Saa and Çetin, 2013), they have not assessed in closed-loop settings. In Saa and Çetin (2012), HMM surpassed CRFs for the classification of EEG signals, but were outperformed by a CRF variant, namely a hierarchical CRF. In contrast, in Saa and Çetin (2013), HMM-based EEG classification accuracy was inferior to the CRF-based one. In Chiappa and Bengio (2003), IOHMMs were found to outperform HMMs for EEG dynamic classification. As both dynamic classifiers performed similarly to their static counterparts (namely, a Gaussian Mixture Model-based Bayes classifier and an ANN), the authors concluded that ANNs surpassed Gaussian mixture model-based generative classification for the considered EEG dataset. It has correspondingly been suggested that multi-state dynamic classifiers may have limited interest for asynchronous decoding (Lotte et al., 2007).

##### 3.2.3.1. Respective relevance of static and dynamic classifiers

In a few papers, the potential advantages of dynamic over static modeling have been investigated in the case of offline data, such as Cincotti et al. (2003) and Delgado Saa et al. (2016). In Cincotti et al. (2003), HMMs were significantly outperformed by ANNs for the classification of right and left hand motor imageries in EEG signals. In Delgado Saa et al. (2016), an extension of CRFs improved the discrimination between finger movements from ECoG signals when compared to LR and sparse linear regression. The interpretability of this result is nevertheless limited, because the considered static and dynamic classifiers did not belong to the same class of models, in contrast with the static-dynamic pairs compared in Chiappa and Bengio (2003).

Finally, post-processing techniques, which rely on *a priori* knowledge about specific characteristics of the user intended effector movement, can be used to improve the movement estimates. When applied on the output of a discrete decoder, they are mainly used to take into account the *a priori* knowledge that fast switches between classes are unlikely. Typical post-processing methods include filtering of the classifier output (Mason and Birch, 2000; Millán and Mouriño, 2003; Bashashati et al., 2007b; King et al., 2015), triggering a state transition after successive identical state estimates only (Townsend et al., 2004; Pfurtscheller et al., 2010), or blocking state transitions for a predefined duration after a performed transition (Townsend et al., 2004; Pfurtscheller et al., 2010).

### 3.3. Continuous decoding

Decoding of continuous dependent variables is mainly performed within the framework MEA- and ECoG-based motor BCI systems, such as kinematic motor BCIs (Hochberg et al., 2006; Collinger et al., 2013; Wodlinger et al., 2015). Continuous dependent variables typically characterize the position or velocity of the effector's endpoint, such as wrist kinetics or kinematics in the case of an upper-limb orthosis (Li, 2014). The use of different classes of models has been explored in BCI studies.

#### 3.3.1. Linear and non-linear regression models

Both linear and non-linear regression models have been applied for kinematic parameter reconstruction from neural signals.

##### 3.3.1.1. Linear regression models

Neural control over prostheses, orthoses, or virtual effectors has been achieved by means of linear models in several online motor BCI studies, both with human (Hochberg et al., 2006; Collinger et al., 2013; Wodlinger et al., 2015) and primate subjects (Taylor et al., 2002; Carmena et al., 2003; Velliste et al., 2008; Suminski et al., 2010; Willett et al., 2013; Williams et al., 2013). Offline trajectory reconstruction has also been performed by means of linear models in several EEG-, ECoG-, and MUA/SUA-based preliminary studies (Bradberry et al., 2010; Koyama et al., 2010b; Liang and Bougrain, 2012; Eliseyev and Aksenova, 2014; Bundy et al., 2016). Linear models rely on the assumption that the dependent variable is a noisy linear combination of the independent variable components; that is, of the neural features:

$$y^{t}=Bx^{t}+\epsilon^{t}$$

where **B** ∈ ℝ^*n*×*m*^ and ϵ^*t*^ ∈ ℝ^*n*^ is the observation noise, and where the neural features *x*^*t*^ can embed a history of instantaneous neural features ${\tilde{x}}^{t}$, i.e., *x*^*t*^ = ${\tilde{x}}^{\left(t+1-\tau_{2}\right):t}$ to exploit neural signal temporal characteristics (Dietterich, 2002; Lotte et al., 2007).

A linear model, namely the Population Vector Algorithm (PVA), has been used for kinematic decoding in several SUA/MUA-driven motor BCI systems (Taylor et al., 2002; Velliste et al., 2008; Collinger et al., 2013; Wodlinger et al., 2015). The PVA is based on the cosine directional tuning model (Georgopoulos et al., 1986), which states that neurons of the motor cortex fire preferentially in one specific direction. The instantaneous firing rate of each neuron is used to weight the corresponding preferred direction.

The use of different identification algorithms has been reported in online and offline motor BCI studies. While Ordinary Least Squares (OLS) corresponds to the maximum likelihood estimator when the measurement noise is Gaussian, the OLS estimator is unstable when the input variable *x*^*t*^ is high dimensional or composed of correlated explanatory features (Friedman et al., 2001). The use of penalized approaches such as pace regression (Kubánek et al., 2009), ridge regression (Suminski et al., 2010; Shanechi et al., 2013; Willett et al., 2013) and sparse linear regression (Williams et al., 2013) has, therefore, been frequently proposed for trajectory model identification and for Ridge regression; for example, outperformed OLS regression in the comparative study drawn in Li et al. (2009) for MUA/SUA-based trajectory reconstruction. Accurate decoding from ECoG high dimensional feature representations has additionally been reported using partial least squares and its variants (Shimoda et al., 2012; van Gerven et al., 2012; Eliseyev and Aksenova, 2014, 2016; Bundy et al., 2016).

##### 3.3.1.2. Non-linear regression models

Linear regression models rely on unrealistic assumptions about information encoding in motor neural signals, whose complexity has been suggested in numerous studies (Scott, 2008). For example, several teams have investigated the use of non-linear models for neural signal decoding (Li, 2014), assuming that *y*^*t*^ = *f*(*x*^*t*^) + ϵ with *f* non-linear. These studies mainly consisted of offline trajectory reconstructions (Kim K. H. et al., 2006; Eliseyev and Aksenova, 2014; Spüler et al., 2016).

Generalized Additive Models (GAM) and Generalized Linear Models (GLMs) constitute one class of non-linear models which interest has been explored for motor BCI systems; for example, for offline trajectory reconstructions from primate ECoG signals in Eliseyev and Aksenova (2014) and Engel et al. (2017). GAMs extend linear models by applying a non-linear function *g*_*i*_ on each component $x_{i}^{t}$ of the independent variable before using a linear model to combine them. In the case of generalized linear modeling, a non-linear function *g*^−1^ is directly applied on the output of a linear filter β, i.e., *y*^*t*^ = *g*^−1^(β*x*^*t*^). The non-linear function *g*^−1^, which is referred to as “link” function, is fit on the training data (Eliseyev and Aksenova, 2014; Engel et al., 2017). A similar approach, namely a cascaded Wiener filter, has been applied on SUA/MUA offline datasets in Flint et al. (2012), Flint et al. (2013), and Scheid et al. (2013).

The application of non-linear regression models such as Support Vector Machine Regression (SVR) (Mehring et al., 2003; Kim K. H. et al., 2006) or ANN models (Sanchez et al., 2002; Hatsopoulos et al., 2004; Kim K. H. et al., 2006; Kim S.-P. et al., 2006) has been additionally proposed for SUA/MUA decoding, and tested in offline preliminary studies or, more recently, in online motor BCI studies (Sussillo et al., 2016). SVR- (Spüler et al., 2016) and Gaussian Processes-based (Wang et al., 2010) trajectory reconstruction has also been reported in ECoG-driven offline BCI studies.

##### 3.3.1.3. Respective relevance of linear or non-linear models for continuous decoding

The findings of several offline preliminary studies are consistent with the idea that non-linear regression models are likely to be more realistic than linear ones for kinematic decoding: linear decoders were outperformed by both GLM and GAM approaches for ECoG signal decoding in monkeys' (Eliseyev and Aksenova, 2014) signals, and by SVR in simulated primate SUA/MUA signals (Kim K. H. et al., 2006). Because non-linear models are more flexible than linear ones and, therefore, more prone to overfit, fine identification procedures were often required for non-linear models proper training. Difficulties were reported for the training of the ANN used in Kim K. H. et al. (2006) for trajectory decoding. They were presented as a possible cause for the superior decoding performance of the SVR, which is yet less flexible than ANNs (Kim K. H. et al., 2006). A specific early-stopping procedure was utilized to prevent overfit during ANN training in Hatsopoulos et al. (2004). The complexity of ANNs' possible structures (e.g., number of layers and number of neurons per layer) additionally makes their optimization time-consuming, which is the reason why proper optimization of the ANN structure was not performed in Hatsopoulos et al. (2004). Under these conditions, linear- and ANN-based trajectory reconstructions from MUA/SUA signals yielded similar results in this study (Hatsopoulos et al., 2004).

Linear models are regularly chosen over their non-linear counterparts in spite of their simplistic assumptions, particularly in the case of MUA/SUA- (Taylor et al., 2002; Velliste et al., 2008; Collinger et al., 2013; Wodlinger et al., 2015) and ECoG-driven (Schalk et al., 2008; Wang W. et al., 2013) online motor clinical BCI studies. Up to 10D- and 3D-control has been achieved by means of linear filtering of MUA/SUA and ECoG signals, respectively (Wang W. et al., 2013; Wodlinger et al., 2015). Linear models have additionally been shown to be reasonably efficient for position, velocity, acceleration, speed and so on in offline decoding (Wang et al., 2007; Bundy et al., 2016; Hammer et al., 2016) and they generally involve simpler training procedures than non-linear models.

Over the course of the last decade, another class of decoders, namely dynamic models, has gained popularity in motor BCIs.

#### 3.3.2. Dynamic models

##### 3.3.2.1. Stochastic state-space models

Linear or GAM-, GLM-, SVR- and generic ANN-based decoders are static regression models; that is, they assume the existence of a (parametric or non-parametric) linear or non-linear model *f* so that *y*^*t*^ ≈ *f*(*x*^*t*^). By contrast, most dynamic models utilized for cursor or prosthesis control in several motor BCIs (Hochberg et al., 2012; Ifft et al., 2013) consider stochastic state-space models; that is,

(1) $$\begin{array}{l} y^{t+1}=g(y^{t})+w^{t},\; \end{array}$$

(2) $$\begin{array}{ll} x^{t} & =h(y^{t})+v^{t}. \end{array}$$

The noise processes *w*^*t*^ and *v*^*t*^ are generally independent and identically distributed sequences of random variables (Krishnamurthy, 2016). The continuous response variable *y*^*t*^ ∈ ℝ^*n*^ is here composed by the trajectory coordinates and derivatives (velocity, acceleration etc.). The transition equation (1) explicitly describes the dynamic of the hidden sequence *y*^*t*^ ∈ ℝ^*n*^ (“movement model” Li, 2014). As expressed in (1), movement models traditionally rely on a first-order Markovian temporal dependencies used to constrain the trajectory smoothness (Brockwell et al., 2004; Koyama et al., 2010b). The dependence between measurements *x*^*t*^ ∈ ℝ^*m*^ and hidden state value *y*^*t*^ ∈ ℝ^*n*^ is described by the emission equation (2), where *v*^*t*^ is the observation noise. As the emission equation models how neural features are generated conditionally to a given trajectory point, state-space models are sometimes referred to as “generative models” (Wu et al., 2002; Gao et al., 2003; Kim S.-P. et al., 2006).

Recursive Bayesian estimation procedures are generally used to infer the hidden trajectory *y*^*t*^ ∈ ℝ^*n*^ from the sequence of noisy measurements *x*^*t*^ ∈ ℝ^*m*^ (Bishop, 2006).

##### 3.3.2.2. Recursive Bayesian estimation: Kalman filter

The Kalman Filter (KF) is a recursive estimation procedure that has been frequently utilized for online and offline trajectory reconstruction. It was first applied for 2D offline hand trajectory decoding from SUA/MUA signals in monkeys (Wu et al., 2002, 2003a), where it was found to surpass linear filtering (Wu et al., 2003a). It has since then provided users with MUA/SUA-based control over prostheses (Hochberg et al., 2012). It has additionally been applied for trajectory decoding from ECoG signals in online and offline studies (Pistohl et al., 2008; Kellis et al., 2012; Marathe and Taylor, 2013; Wang et al., 2013b). KF applies to linear Gaussian state-space models (Bishop, 2006); that is, to state-space models with linear emission and transition models associated with Gaussian noises.

After training, typically performed using Ordinary Least Squares (Wu et al., 2002), the KF issues the estimate ${\hat{y}}^{t}=E\left(y^{t}|x^{1:t}\right)$.

##### 3.3.2.3. Alternative recursive estimation procedures

To the best of our knowledge, dynamic modeling of ECoG data has been restricted to Gaussian state-space models; that is, Kalman filtering procedures. However, further investigations have been carried out to ascertain the interest of non-linear and/or non-Gaussian state-space modeling of MUA/SUA data. While non-linear and/or non-Gaussian state-space representations integrate more realistic emission and noise models (e.g., Poisson noise for spiking counts), the associated trajectory estimation procedures are often approximate and/or computationally expensive (Koyama et al., 2010a) [e.g., Unscented Kalman Filter (UKF) in (Li et al., 2009, 2011; Ifft et al., 2013), particle (Brockwell et al., 2004), point-process or Laplace-Gaussian Filtering (LGF) (Velliste et al., 2014) in the case of Poisson noise].

The relevance of non-linear emission model has been studied for MUA/SUA offline decoding in Gao et al. (2003). The emission model was modeled using linear models, GLM or GAM associated with Poisson noises (Gao et al., 2003). Non-linear models, and particularly GAM-based emission models, were found to improve the quality of trajectory estimation. In Koyama et al. (2010b), KF and the LGF (i.e., a procedure for non-linear emission models and Poisson noise) performed similarly for offline trajectory reconstruction from primate SUA/MUA signals. An additional closed-loop study suggested a slight superiority of the LGF over the KF (Koyama et al., 2010b). Analogously, the UKF proposed in Li et al. (2009) surpassed traditional KF for a task of trajectory reconstruction from MUA/SUA signals.

##### 3.3.2.4. Alternative dynamic models

The use of dynamic ANN (e.g., recurrent neural networks) has recently been reported for MUA/SUA-based kinematic decoding (Sussillo et al., 2016).

##### 3.3.2.5. Static vs. dynamic models for continuous decoding

Over the last few years, dynamical models have emerged as a promising and efficient alternative to static (typically linear) models (Srinivasan et al., 2007; Li, 2014). Since its first application in 2002, the Kalman filter and its variants have been increasingly applied for both online and offline SUA/MUA decoding (Wu et al., 2002; Hochberg et al., 2012; Aggarwal et al., 2013). Because of the deterrent computational burden of its variants, recursive estimation during closed-loop experiments has mainly been achieved via Kalman Filtering (Li et al., 2009). While the respective performance of Wiener and Kalman filters appeared to depend on the decoding task at hand in Kim S.-P. et al. (2006), a few systematic comparisons of static and dynamic models have given a steady ground to the popularity of the Kalman filter: static linear models were outperformed by dynamical ones for open-loop (Aggarwal et al., 2009) and/or closed-loop SUA/MUA decoding (Koyama et al., 2010b). The KF embedded approach for smoothing has been identified as particularly efficient in both open- and closed-loop studies (Koyama et al., 2010b).

By contrast, the respective suitability of static and dynamic continuous models for ECoG decoding is still unclear. ECoG-based neural control has been achieved by means of a linear decoder both in non-human primates (Williams et al., 2013) and human subjects (Wang W. et al., 2013) (2D and 3D effector control, respectively). The use of linear decoding has also been reported for ECoG offline trajectory reconstruction (Schalk et al., 2007; Chao et al., 2010; Liang and Bougrain, 2012; Shimoda et al., 2012; Hammer et al., 2013; Nakanishi et al., 2013; Wang W. et al., 2013; Williams et al., 2013; Hotson et al., 2014; Bundy et al., 2016). Up to 7DoF have been reconstructed in offline feasibility studies led on primate (Chao et al., 2010; Shimoda et al., 2012) and human subjects (Schalk et al., 2007; Nakanishi et al., 2013). Meanwhile, Kalman filtering has permitted to reconstruct 2D kinematic parameters from ECoG signals (Pistohl et al., 2008; Kellis et al., 2012; Marathe and Taylor, 2013; Wang et al., 2013b). In a comparative study performed on ECoG data (Eliseyev and Aksenova, 2016), static models outperformed Kalman filtering for the reconstruction of kinematic parameters from high-dimensional time-space-frequency feature representations. One reason for these findings could be a lesser relevance of generative approaches for high-dimensional ECoG data. In another study led on ECoG data (Marathe and Taylor, 2013), Kalman-based cursor control was more precise than linear-decoder-based control.

Post-processing of a continuous movement estimate is generally used with the aim of improving the smoothness of the corresponding decoded trajectory, whose impact on control has been investigated in Marathe and Taylor (2015). The improvement of other trajectory characteristics requires the existence of an *a priori* trajectory model, which is seldom available. A complex *a priori* finger trajectory model based on a switching non-linear dynamic model was for example built in Wang et al. (2011). This model in particular integrated *a priori* knowledge about the succession of rest, flexion and extension states and about the maximal amplitude of finger movements. The switching post-processing model was applied on the output of a linear decoder fit on both idle and active samples, and permitted both to support idle state and accurately decode multi-limb trajectories (Wang et al., 2011).

## 4. Discussion

The many online and offline studies cited in the present review attest to the efforts that have been made in the last few years toward designing accurate decoders for motor BCI systems. Decoding and training strategies have been confronted, and modeling approaches have been compared in offline and online studies so as to establish the respective relevance of decoding solutions. Yet, in spite of sustained efforts and of proofs of concept performed in laboratory environments (Collinger et al., 2013; Wodlinger et al., 2015), motor BCIs have not yet been deployed for everyday use (Mak and Wolpaw, 2009). This lack of clinical motor BCIs contrasts with the few communication or environmental control BCI systems which have been commercialized (Mak and Wolpaw, 2009), e.g., EEG-driven spellers (G.tex, 2016). This is due to the technical difficulties encountered to meet the particularly demanding features required for motor BCIs to benefit patients in their everyday life. While some of these technical obstacles concern the acquisition system, such as the development of safe and chronic acquisition systems which is still under progress, several challenges are more specifically associated with the BCI transducer. The challenges related to the design of clinical-compatible motor BCI transducers include high decoding accuracy, chronicity, asynchronicity, multiple DoFs and/or multi-limb decoding.

### 4.1. Accuracy

The high fidelity of the transducer's estimates to the user's intentions is generally deemed to be crucial for motor BCI systems. It has been shown that an accurate extraction of kinematic parameters from the neural signals is required for upper-limb motor BCI systems to enable patients to interact with their environment, such as perform efficient reaching movements (Marathe and Taylor, 2011). Most of the transducers that are mentioned in the present review were designed with the implicit or explicit objective of providing highly accurate estimates of the user's intended movements during periods of active effector control. Correlations as high as 0.8 or 0.9 have been obtained between real and MEA-based offline estimates of upper-limb movements (Li et al., 2009; Vargas-Irwin et al., 2010).

Yet, despite the noteworthy algorithmical developments and refinements which have resulted in considerable progress in the reported decoding accuracies, the ability of motor BCI users to interact with their environment remains limited. While a normally functioning upper limb is associated with a score of 27 in the Action Research Arm Test (ARAT), scores ranging from 14 to 17 and or averaging around 15.5 have been reported after training of tetraplegic patients in two forefront studies (Collinger et al., 2013; Wodlinger et al., 2015). The control complexities obtained with semi- or non-invasive acquisition technologies (i.e., with ECoG or EEG arrays), for their part, have not yet equalled the performances achieved in these two MEA-driven motor BCI systems. Similarly, even if they were to be maintained in closed-loop experiments, the high accuracies reported in offline studies (Li et al., 2009; Vargas-Irwin et al., 2010) may not be sufficient to permit users to proficiently control upper-limb protheses or orthoses. Therefore, decoding accuracy is still an obstacle that prevent motor BCIs being of use to severely motor impaired patients.

### 4.2. Chronicity

While chronic signal acquisition remains a critical problem for invasive motor BCI systems because of the biocompatibility issues associated with MEAs (Mak and Wolpaw, 2009), efficient chronic decoding is no less challenging. Invasive MEA-based motor BCI systems regularly require daily recalibrations (Hochberg et al., 2012; Wodlinger et al., 2015; Bouton et al., 2016) which may be cumbersome for long-term utilizations. These recalibrations are made necessary by the instability of neural motor representations, which have been disclosed for MUA/SUA in Rokni et al. (2007). Similarly, adjustments to the parameters of transducers embedded in non-invasive or semi-invasive BCI systems seem beneficial. Several sources of signal instabilities have, for example, been disclosed in the case of EEG signals, such as instabilities caused by muscle tension, environmental noise, attention, mood or fatigue (Mladenovic et al., 2017).

While both efficient adjustment of the transducer parameters and decoder robustness to neural variability (Sussillo et al., 2016) are desirable, they remain challenging tasks. Adaptive algorithms are one of the paths currently explored by the BCI community (Gürel and Mehring, 2012; Merel et al., 2013; Zhang and Chase, 2013).

### 4.3. Asynchronous control

Another major issue for motor BCI clinical applications is the ability to provide users with asynchronous control over the effector (Graimann et al., 2009). Most motor clinical trials have been completed using a synchronous protocol, i.e., user intentions were not processed outside predefined, cued windows (Hochberg et al., 2006; Wodlinger et al., 2015). Given that the deployment of synchronous BCI systems requires the presence of an operator to switch the system on and off, the impact of the BCI system on users' independence is limited. Potential BCI users, however, express a strong desire for stand-alone BCI systems (Blabe et al., 2015). Because asynchronous (or self-paced) BCI transducers are continuously available to BCI users (Graimann et al., 2009), they potentially correspond to stand-alone systems. Asynchronicity is, therefore, an essential feature for practical motor BCIs (Wolpaw et al., 2002).

In asynchronous mode, users generally alternate between periods of Intentional Control (IC) and of No-Control (NC), during which they do not intend to use the BCI system (Mason et al., 2006). The limitation of erroneous activations of the BCI system during NC states is all the more important when users of motor BCIs physically interact with the effector (e.g., an orthosis) in contrast to BCIs based on the control of a virtual effector (e.g., a cursor on a computer screen). Because false activations are likely to be particularly disturbing and stressful to users, NC support (i.e., the prediction of neutral values during NC states) is highly desirable for motor BCIs (Leeb et al., 2007).

NC support has first been studied for brain-switches; that is, BCI systems which integrate NC detection into discrete decoders by considering and detecting a NC class (Bashashati et al., 2007b; Pfurtscheller et al., 2010; Solis-Escalante et al., 2010). Linear discriminant analysis (Pfurtscheller et al., 2010), support vector machine (Solis-Escalante et al., 2010), logistic regression (Blokland et al., 2014) or dynamic classifiers such as hidden Markov models or HMM variants (Kemere et al., 2008; Gouy-Pailler et al., 2009; Hotson et al., 2016) and conditional random fields (Hasan and Gan, 2011; Delgado Saa et al., 2016) are some of the classifiers which use has been reported to distinguish NC states from motor imageries (Gouy-Pailler et al., 2009) or movement execution (Delgado Saa et al., 2016).

Although asynchronous control has been considered in a few motor clinical BCIs relying on discrete decoders, such as in Hotson et al. (2016), NC states were not supported in the majority of biomimetic kinematic motor BCIs, which were deployed using synchronous paradigms (Hochberg et al., 2006, 2012; Wodlinger et al., 2015). The integration of NC support into kinematic decoders has only been partially addressed in the literature. Although generic linear static and dynamic models are favored for motor BCI kinematic decoders, they usually fail to output zero-velocity (neutral) estimates when they are used for asynchronous decoding and are applied to NC states (Chao et al., 2010; Shimoda et al., 2012; Velliste et al., 2014). Several different decoding strategies have been used to integrate NC support into biomimetic kinematic neural signal decoders. While most of them were validated with offline analyses, the integration of NC support into a kinematic decoder was nevertheless utilized for asynchronous control of a robotic arm in monkeys in Suway et al. (2013).

A first approach to integrate NC support into biomimetic kinematic decoders, namely post-processing, has been explored for both SUA/MUA (Aggarwal et al., 2013; Velliste et al., 2014) and ECoG signals (e.g., Wang et al., 2013b) decoding. This consists in overwriting the output of the single kinematic model with null-velocity (neutral) estimates when a NC state is detected by a discrete NC/IC decoder. Different pairs of discrete-continuous decoders have been considered. Kalman filters and (non-Gaussian) variants have been gated by LDA in SUA signals in Velliste et al. (2014) and Aggarwal et al. (2013) or Bayes classifier in ECoG signals (Wang et al., 2013b), i.e., their output was overwritten with neutral values when an independent classifier detected a NC state (Aggarwal et al., 2013; Wang et al., 2013b; Velliste et al., 2014). Finally, a dynamic post-processing model permitted to integrate NC support into a finger kinematic decoder in Wang et al. (2011).

Another approach to asynchronous decoding consists in embedding NC support into the decoder. While the use of generic non-linear models has been considered in a few studies (e.g., GLM or GAM Eliseyev and Aksenova, 2014), the most popular decoding approach consists in switching between continuous models. Switching models rely on a latent discrete variable to introduce state-specific non-linearities into a generic continuous decoder (Wood et al., 2005; Srinivasan et al., 2007; Bundy et al., 2016; Schaeffer and Aksenova, 2016), where some of states are associated with NC periods. Both static (Williams et al., 2013; Bundy et al., 2016) and dynamic (Wood et al., 2005; Srinivasan et al., 2007) switching models have been considered for asynchronous mono-limb decoding from ECoG (Williams et al., 2013; Bundy et al., 2016) or MUA/SUA (Wood et al., 2005) signals. In the case of dynamic switching models (e.g., the switching particle filter; Wood et al., 2005), the value of the latent variable is used to switch between observation and/or transition models. The transition model associated with NC states explicitly takes into account the fact that null-velocity estimates are expected during NC states.

However, despite the clear needs and considerable efforts, it is likely that the level of false positive activation generally reported in literature (e.g., Delgado Saa et al., 2016; Hotson et al., 2016) is still too high for practical asynchronous motor BCI systems (Fatourechi et al., 2008).

### 4.4. Multi-limb control

Although it may improve the daily lives of severely impaired patients (e.g., patients with tetraplegia), multi-limb decoding has been scarcely explored by the BCI community. While numerous daily life tasks require bimanual movements (Swinnen and Wenderoth, 2004), bimanual neural control has only been reported over virtual effectors (Ifft et al., 2013). Similarly, combined lower- and upper-limb control has yet to be thoroughly explored by the BCI community. The integration of multi-limb effector control into motor BCIs has only been considered in a few studies focusing on upper limbs (Hochberg et al., 2012; Ifft et al., 2013; Wodlinger et al., 2015; Bouton et al., 2016). Both parallel and sequential multi-limb control have been investigated.

Parallel control consists in decoding and allowing the movement of several effector at once, such as simultaneous movements of hand and arm prostheses or orthoses. This is generally performed by applying several limb-specific models on neural features (Hochberg et al., 2012; Wodlinger et al., 2015). Limb-specific models are trained jointly or independently.

In the case of sequential control, , one limb only is active at each time moment. When several limb-specific models are applied on neural features, the activation of one limb can result in residual movements of the other limbs. Such noisy outputs were, for example, observed in Nakanishi et al. (2014), where the displacement of a finger resulted in small-amplitude movements in the estimations of the other fingers' position. A switching model was thus considered for sequential asynchronous multi-finger decoding in Flamary and Rakotomamonjy (2012). One linear model was devoted to each finger, and applied when deemed appropriate by a multi-class discrete decoder. These switching models intrinsically prevent parallel activations as only one active limb model is chosen at each instant.

The extension of mono-limb asynchronous decoding to multi-limb asynchronous control is an additional challenge for multi-limb BCI systems. The switching models designed for asynchronous control can readily be applied for asynchronous parallel multi-limb control. They can additionally be extended for asynchronous sequential multi-limb neural control by considering one or more states per limb and one state for NC period.

### 4.5. Summary on the current progress and conclusion

While EEG-based motor BCIs have the significant advantage of being safe, a long training process is generally necessary before the user is able to adapt to the mental-task decoder they embed. To date, the corresponding complexity of control additionally remains inferior to that associated with invasive and semi-invasive BCIs. 2D-control over a robotic arm with four possible directions (Hortal et al., 2015), 3D synchronous control (LaFleur et al., 2013), or 2D control decomposed into sequences of 1D movements (Bhattacharyya et al., 2015) have been reported in EEG-based motor BCI systems.

The implantation of invasive or semi-invasive acquisition systems (MEA, ECoG) is still associated with biocompatibility and chronicity issues. However, their comparatively higher informative content may make of them a promising alternative to EEG for highly accurate, multiple DoF and multi-limb control (Lebedev and Nicolelis, 2006). The feasibility of 3D (Hochberg et al., 2012), 7D (Collinger et al., 2013), and 10D (Wodlinger et al., 2015) neural control over a robotic arm has been demonstrated in recent MEA-based studies. The integration of multi-limb effector control into motor BCIs has particularly been considered in a few studies (Hochberg et al., 2012; Ifft et al., 2013; Wodlinger et al., 2015; Bouton et al., 2016). In Hochberg et al. (2012), both sequential or parallel MEA-based control over an upper-limb prosthesis endpoint and a prosthetic hand were achieved by users with tetraplegia. Parallel control over the wrist and hand of a robotic arm was additionally reported in Wodlinger et al. (2015). Bimanual control has only been reported over virtual effectors (Ifft et al., 2013). While these studies suggest the relevance of MEA acquisition systems and of the reported decoder structure and training strategy, the issues pertaining to the MEA invasiveness—namely, safety and chronicity (Vouga et al., 2017)—are to date only partially addressed.

While ECoG arrays hold promise of chronic and stable signal acquisition (Costecalde et al., 2017), the reported ECoG-driven motor BCIs generally relied on mental-task decoders (Schalk et al., 2008; Wang W. et al., 2013; Fifer et al., 2014; Kapeller et al., 2015) and did not permit users to achieve complex effector control. Control over a set discrete commands was achieved using ECoG signals in Fifer et al. (2014) and Hotson et al. (2016), and 3D (Wang W. et al., 2013), 2D (Schalk et al., 2008), and 1D control (Vansteensel et al., 2010; Leuthardt et al., 2011) has been reported in a few studies. While kinematic control has not yet been completed in human subjects, 2D kinematic control was accomplished by monkeys in Marathe and Taylor (2013). ECoG-based multi-limb control has mainly been considered in the case of multi-finger offline trajectory reconstruction (Kubánek et al., 2009; Wang W. et al., 2009; Acharya et al., 2010; Flamary and Rakotomamonjy, 2012; Liang and Bougrain, 2012; Wissel et al., 2013; Nakanishi et al., 2014; Delgado Saa et al., 2016). Individual finger ECoG-based control was restored in Hotson et al. (2016). To date, the degree of complexity achieved with ECoG-driven control is consequently surpassed by those reported for MEA-based BCIs. However, synchronous protocols have mainly been considered and studies on ECoG-based effector chronic control are still lacking. Therefore, the proof that chronic asynchronous ECoG control over multi-limb multi-DoF effectors is feasible remains to be established. The development of chronic and fully implantable signal acquisition systems compatible with clinical applications remains critical for invasive motor BCIs systems because of the associated biocompatibility issues and of additional constraints such as the patients' aspiration to wireless signal transmission. The utilization of fully wireless neural signal acquisition systems has recently been reported (Kohler et al., 2017). For example, the wireless 64-channel ECoG implant WIMAGINE®, which has been designed for long-term signal acquisition (Mestais et al., 2015) has recently been developed. This is able to record brain activity on 64 low noise channels and to wirelessly transmit data to a computer for further analysis. It additionally complies with Active Implantable Medical Devices standards. CLINATEC has more specifically developed an ECoG-based BCI platform dedicated to chronic clinical use (Eliseyev and Aksenova, 2014), with the goal of bringing the proof of concept that it is feasible for a tetraplegic subject to control complex effectors (e.g., a 4-limb exoskeleton) after training thanks to the decoding of his cortical brain electrical. This demonstration is the aim of a 5-year clinical trial approved by the French competent authorities (ClinicalTrials.gov, 2016). These implants may make ECoG a viable alternative to MEA for motor BCI systems.

Despite the many examples of impressive progress, there is still room for considerable improvements in the design of transducers able to maintain high neural signal decoding performance out of laboratory environments. Motor BCI systems may particularly benefit from the transfer of advances from the area of machine learning to the field of neural signal decoding.

## Author contributions

M-CS and TA wrote the manuscript. Part of this work is derived from the Ph.D. of M-CS previously published in Schaeffer (2017).

### Conflict of interest statement

The authors declare that the research was conducted in the absence of any commercial or financial relationships that could be construed as a potential conflict of interest.
